# Anisotropic persistent random walk model simulates T-cells migration over curved landscapes

**DOI:** 10.1038/s41598-025-02804-3

**Published:** 2025-06-04

**Authors:** Gildas Carlin, Ian Manifacier, Dang Khoa Cao, Laurent Pieuchot, Valeriy Luchnikov, Jean-Louis Milan

**Affiliations:** 1 Aix Marseille Univ, CNRS, ISM, Marseille, France; 2 Aix Marseille Univ, APHM, CNRS, ISM, Sainte-Marguerite Hospital, Institute for Locomotion, Department of Orthopaedics and Traumatology, Marseille, France; 3 Université de Haute-Alsace, CNRS, IS2M UMR 7361, F-68100 Mulhouse, France; 4 Univ Gustave Eiffel, Aix Marseille Univ, LBA, F-13016 Marseille, France

**Keywords:** Curvature, Cell migration, Curvotaxis, In silico model, Biophysics, Cell biology

## Abstract

Cell migration is an important cellular process to study, as it plays a fundamental role in tissue structuring and development, while abnormal cell migration may be the cause of certain diseases. Among the known factors influencing cell migration, substrate curvature is one, with cells naturally moving towards concave areas while avoiding convex ones. The underlying causes of migration guidance by curvature remain unclear, and in particular, the way in which cell persistence is affected is still not well understood. We introduce an anisotropic persistent random walk model which includes cell heterogeneity to simulate T-cell migration across various corrugate landscapes. We compared the trajectories generated by the model with in vitro T-cells trajectories over the same topographies. The model accurately captures key features of cell trajectories on flat surfaces as well as on curved surfaces, such as a directional bias toward concave regions. The model also reveals a superdiffusive behavior on curvature, demonstrating more efficient movement compared to flat surfaces. The anisotropic randomness incorporated in the model appears as a critical feature which shapes T-cells persistence mechanisms by increasing cellular activity in the axis of concave valleys and promoting migration towards concave areas.

## Introduction

Cell migration is a key process in the formation and the tissues organization which occurs during the embryo’s development, wound healing, immune response and major healthcare issues such as cancer metastasis and diabetes^1–9^. Cell motility is regulated by inner-cell signaling events^10,11^ excited or inhibited by external stimuli: cell-to-cell interactions, chemical or mechanical cues^6,12–15^. Shellard and Mayor^16^ provide a review of known cues guiding cell migration. Among these, curvotaxis—directed cell migration over curved surfaces—emerges as a novel topographic cue influencing the migration of individual cells^17,18^. In vivo, curvature emerges from cell packing and tissues growth^19,20^. In addition, curvotaxis can also be harnessed to design smart substrates or scaffolds for applications in cell and tissue engineering^21–23^.

In vitro, one of the first cell migration experiments over anisotropic curved surface was developed by Song et al.^24^. They cultured T-cells over wavy surfaces of various curvatures across scales. They observed that T-cells are located mostly in the concave areas and migrate along valleys, this phenomenon is more important when the curvature increases. They found that crossing a convex requires myosin-II contractility; and stronger focal adhesions increase cell response to curvature. In the same line, Pieuchot et al.^25^ brought to light the role of the cell nucleus in curvotaxis. They cultured mesenchymal cells and fibroblasts over a hills-and-valleys surface and observed that these two cell lines migrate preferentially in the valleys avoiding convex hills; after one day of culture, cells tend to stabilize in the concave valleys.

The use of numerical models alongside in vitro studies has increased in recent years, as it complements experimental approaches by making it easier to test assumptions, explore a wide range of conditions, and optimize experimental setups. Our group has developed a mechanical model of single cell^26–28^ that represents its contractile cytoskeleton by ensembles of non-penetrable rigid particles linked by springs and cables. This model successfully reproduced the in vitro curvotaxis phenomenon reported in Pieuchot et al.^25^ and suggests a physical explanation of curvotaxis: forces generated by actin-myosin network contractions at adhesion sites on the surface are transmitted to the nucleus, causing it to compress and move within the cell toward regions of greatest concavity. From the inner-cell nucleus displacement results a bias toward concave areas. We recently extended this model to dynamic curvotaxis^28^, i.e. a wave crosses the surface, which provides a proof of concept that curvature can impose a direction of migration over large distances.

Alternative approaches have been developed to simulate cell migration guided by curvature. Phase-field models^29,30^ examine actin-myosin network dynamics and successfully reproduces cell alignments similar to those observed on sinusoidal wavy surfaces^29^. Additionally, the vesicle model of Sadhu et al.^31,32^ simulates the lamellipodia dynamics, suggesting that curvotaxis is a mechanism of membrane bending energy minimization. Cell shape dependence on curvature has also been modeled with qualitative agreements^33^. Effects of curvature are also observable at the multicell and epithelium scale, as concave favors the tissue development^34,35^. Epithelium models have been developed considering the tissue as an active continuous gel^36,37^ or by modeling each cell^38–40^. By incorporating curvature metrics in the equations, some models support the coupling between cells development and substrate curvature.

Most of the previously mentioned models reproducing cell migration on curved substrates are essentially deterministic. In the particles model^26–28^ developed by our group, randomness occurs both during the particles generation at the beginning of the simulation and within the resolution algorithm, which is partially stochastic. However, the simulation remains largely deterministic and cannot generate random trajectories. In Sadhu et al.^31^, randomness is added in vertex displacement during the simulation so there is fluctuations in resulting trajectories but the model is still mostly deterministic.

In vivo cells exhibit erratic trajectories which are not subject to one cue and cannot be entirely captured by deterministic formulations^41^. Cells also persist, i.e. they are able to maintain a cap, and to our knowledge the effect of curvature on persistence has not been modeled. Therefore, we need a model that incorporate effects of curvature on persistence. The persistence ability is measurable through autocorrelation metrics such as the VACF, so the model needs also to include randomness to capture the cell liveliness and compute population relevant metrics.

The Persistent Random Walk (PRW) model is a widely used formulation to recreate cell migration and its associated randomness^41–44^. The cell is modelized by a material point and its dynamic is driven by Newton’s second law. Among the simplest formulations, the Ornstein–Uhlenbeck process (OU process) considers two forces. First, a deterministic force describing the cell ability to persist in a given direction^42^. The second force represents random fluctuations caused by the unpredictable nature of the intracellular signaling mechanisms. Due to inherent stochastic fluctuations, simulations are conducted for a population of cells, and statistical analyses are performed on the resulting trajectories, following standard practices commonly employed by experimentalists.

In this paper, we introduce an anisotropic PRW model with cell heterogeneity where cell polarity is generated following an OU process. We describe the model as anisotropic because cellular behavior varies depending on the axis, and we account for cell heterogeneity: each cell in the simulated population is assigned individual parameters values, which are derived from experimental data by following and adapting the protocol outlined by Wu et al.^45,46^. We numerically simulated T-cell experiments over wavy surfaces with various curvatures and compared our simulated trajectories with the experimental trajectories^24^ kindly provided by the authors. The PRW model has been tested over both flat and curved surfaces, and captures experimental observations such as the directional bias toward the concave areas and cell velocity distribution. We also computed time functions, the mean-squared displacement and the velocity autocorrelation function which were not assessed in the experimental paper. We observed that T-cell migration on curved surfaces exhibits characteristics of anomalous diffusion, which is accurately captured by the model. Additionally, the anisotropic randomness which models cellular activity increases with curvature in the direction of concave valleys which promotes migration in this direction.

## Methods

### Surface curvature

**Fig. 1:**
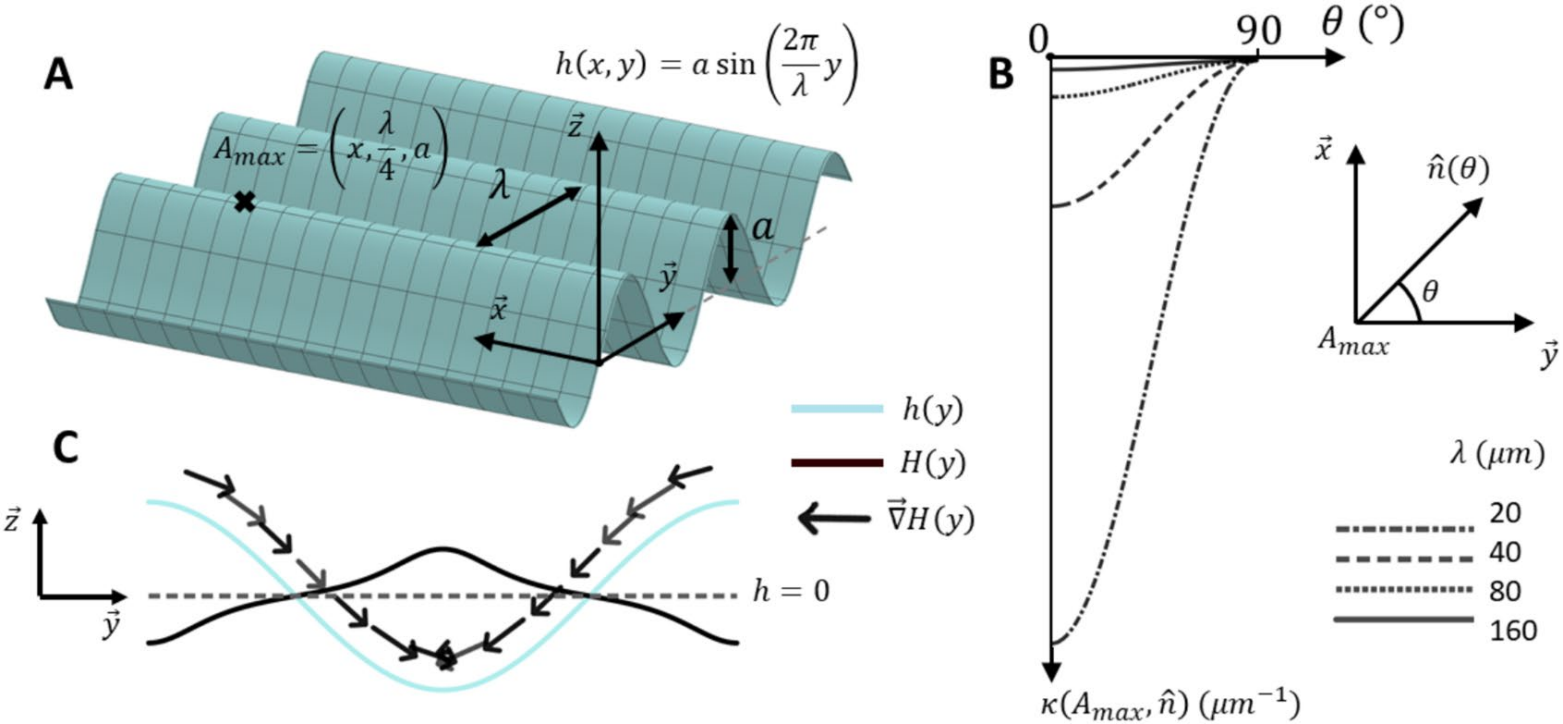
(**A**) Sinusoidal surface with amplitude $a$ and wavelength $\lambda$. The point ${A}_{max}$ located at one of the surface maxima is the reference point in (**B**) to calculate directional curvature $\kappa$. (**C**) Evolution of mean curvature $H$ over the surface. The vector $\overrightarrow{\nabla }H$ points toward the surface minimum. The norm of $\overrightarrow{\nabla }H$ has been expended and kept constant for figure readability.

We reproduced numerically the experiments of Song et al.^24^ and considered the same sinusoidal surfaces with constant amplitude $10$ µm and multiples wavelengths 20, 40, 80 and 160 µm. The model was running over a wavy sinusoidal surface characterized by an amplitude $a$ and a wavelength $\lambda$ (Fig. 1.a). We measure surface curvature as follows. Let $M$ be a point belonging to the surface in an orthogonal frame $\left(O,\overrightarrow{x},\overrightarrow{y},\overrightarrow{z}\right)$, its coordinates are $\overrightarrow{OM}=\left(x,y,h\left(x,y\right)\right)$ in which $h$ is given by Eq. (1),

1 $$h\left( {x,y} \right) = a\,\sin \left( {\frac{2\pi }{\lambda }y} \right).$$

Generally used curvature metrics are intrinsic gaussian curvature, extrinsic mean curvature $H$ or directional curvature $\kappa$^17,18,20,36,47^. Here, we additionally used the vector gradient of mean curvature $\overrightarrow{\nabla }H$. These quantities are standard differential geometry results^48^. The tangent vectors to the surface at the point $M$ are expressed ${\overrightarrow{e}}_{\mu }={\partial }_{\mu }\overrightarrow{OM}$ with $\mu \in \{x,y\}$. The unit-length normal vector to the surface is given by ${\overrightarrow{e}}_{z}=\frac{{\overrightarrow{e}}_{x}\wedge {\overrightarrow{e}}_{y}}{\Vert {\overrightarrow{e}}_{x}\wedge {\overrightarrow{e}}_{y}\Vert }$, and ${\left\{{\overrightarrow{e}}_{\mu }\right\}}_{\mu \in \{x,y,z\}}$ is a local basis at the point $M$. Note that ${\overrightarrow{e}}_{x}$ and ${\overrightarrow{e}}_{y}$ are not unit-length vectors. The directional curvature $\kappa$ at the point $M$ in the direction of a unit-length tangent vector $\widehat{n}$, is given by $\kappa \left(M,\widehat{n}\right)= {\widehat{n}}^{T}\cdot {\underline{\underline{K}}}\left(M\right)\cdot \widehat{n}$ expressed in µm^−1^, with ${\underline{\underline{K}}}$ the curvature tensor where the components ${K}_{\mu \nu }$ are expressed ${K}_{\mu \nu }\left(M\right)= {\overrightarrow{e}}_{z}{\left(M\right)}^{T}\cdot {\partial }_{\mu }{\overrightarrow{e}}_{\nu }(M)$ with $\mu ,\nu \in \left\{x,y\right\}$; the $T$-exponent denotes the transposition.

Figure 1b shows the variation of $\kappa$ at $M={A}_{max}$ when the direction of $\widehat{n}=\left(\mathrm{sin}\theta , \mathrm{cos}\theta \right)$ is changing. When $\widehat{n}$ points toward $\overrightarrow{y}$ ($\theta =0^\circ$), the curvature is at its minimum then collapses to zero when $\widehat{n}$ points toward the $x$-axis ($\theta =90^\circ$). As expected, short wavelengths increase curvature.

We define the mean curvature $H$ expressed in µm^−1^ by Eq. (2),

2 $$H\left( M \right) = \frac{1}{2}\underline{{\underline {g} }}^{ - 1} \left( M \right):\underline{{\underline {K} }} \left( M \right),$$

with “:”, the double dot product and ${\underline{\underline{g}}}$ the metric tensor whose components are defined as ${g}_{\mu \nu }\left(M\right)= {\overrightarrow{e}}_{\mu }(M)\cdot {\overrightarrow{e}}_{\nu }(M)$ with $\mu ,\nu \in \left\{x,y\right\}.$ The “− 1” exponent denotes the inverse matrix. It enables to calculate the gradient of mean curvature $\overrightarrow{\nabla }H\left(M\right)=\left({\partial }_{x}H\left(M\right), {\partial }_{y}H\left(M\right)\right).$ The latter points toward the direction of greatest increase in curvature, i.e. the concave areas. Figure 1.c shows the evolution of the mean curvature $H$ and the gradient of mean curvature $\overrightarrow{\nabla }H$ applied to the sinusoidal surface. See Supplementary Information for expression of $\overrightarrow{\nabla }H$.

### The PRW model

In Vassaux et al.^27^, authors assumed that the nucleus guide the cell towards concave areas, in concordance with experiments^25^. The migration is modelled by creating a bias on the leading lamellipodium which is conditioned by the intracellular nucleus movements towards concave. It has been observed experimentally that focal adhesion (FA) density and properties varies with curvature: FA located on concave tend to be more stable and are subject to higher tension than those on convex^25^. We assume these curvature-induced structural changes modify the cell polarity. In our model, we do not distinguish cell structures involved in cell migration; the resulting action of cell steering and motor structures on the migration is summarized by cell polarity ${\boldsymbol{p}}$. We assume that polarity is following an anisotropic Ornstein–Uhlenbeck process (OU process) (Eq. 3),

3 $$\frac{{{\mathrm{d}}p_{\mu } }}{{{\mathrm{d}}t}} = - \frac{{p_{\mu } }}{{{\Pi }_{\mu } }} + \sqrt {\frac{{S_{\mu }^{2} }}{{{\Pi }_{\mu } }}} \eta \left( t \right),\quad \mu \in \left\{ {x,y} \right\}.$$

Because the surface is anisotropic, the OU process is decomposed in two principal directions corresponding to the principal curvatures, i.e. the eigenvalues of ${\underline{\underline{K}}}$. In the case of a sinusoidal surface, these directions are aligned with $\overrightarrow{x}$ and $\overrightarrow{y}$. The OU process parameters are ${S}_{\mu }$ and ${\Pi }_{\mu }$, respectively the speed in µm/s and the persistence in seconds^42,45^. In a way, $\Pi$ is a persistence inertia, which causes a resistance to cell persistence variation during cell motion and $S$ is perturbations to persistence. The stochastic process simulates randomness in cell behavior as noise: $\eta$ is a Wiener process of variance ${S}_{\mu }^{2}/{\Pi }_{\mu }$.

Polarity is a quantity very similar to the cell velocity ${\boldsymbol{v}}$: it shares the same units and it emerges from cellular activity. However, cell motion is subject to external stimuli, curvature in our case, so the resulting cell velocity is not always equal to its polarity. Surface curvature seems to act both on cell speed and cell direction of migration^17,24,25^, so we decompose the velocity intensity and the velocity direction,

4 $${\boldsymbol{v}} = f\left( {\kappa ,\user2{ p}} \right)\hat{\user2{w}}\left( {\kappa ,{\boldsymbol{p}}} \right),$$

with $f$ and $\widehat{{\boldsymbol{w}}}$ respectively the norm and the direction of ${\boldsymbol{v}}$ which are functions of the curvature $\kappa$ and the polarity ${\boldsymbol{p}}$.

When cells are constraint to migrate in a concave, they exhibit trajectories similar to those observed on flat and they are not faster^49^. On the contrary, when cells migrate on a convex, their nuclei accelerates^25^ which indicates an excited state. This excitation could emerge from an asymmetric focal adhesion density around the cell in parallel of stress fibers reorientation toward the cell nucleus^25^. So, we choose,

5 $$f\left( {\kappa ,{\boldsymbol{p}}} \right) = ||{\boldsymbol{p}}|| + \left\{ {\begin{array}{*{20}l} {\gamma \left| {\kappa \left( {{\boldsymbol{r}}, \hat{\user2{p}}} \right)} \right| ||{\boldsymbol{p}}}|| \hfill & {\quad {\mathrm{if}}\, \kappa \left( {{\boldsymbol{r}}, \hat{\user2{p}}} \right) \le 0} \hfill \\ 0 \hfill & { \quad {\mathrm{if}}\, \kappa \left( {{\boldsymbol{r}}, \hat{\user2{p}}} \right) > 0} \hfill \\ \end{array} } \right..$$

$\gamma$ is a constant homogeneous to a length and $\hat{\user2{p}} = {{\boldsymbol{p}} \mathord{\left/ {\vphantom {{\boldsymbol{p}} {\left\| {\boldsymbol{p}} \right\|}}} \right. \kern-0pt} {\left\| {\boldsymbol{p}} \right\|}}$ denotes the unit-length polarity vector. ${\boldsymbol{r}}$ is the cell position. In Manifacier et al.^28^, a linear relation between cell speed and curvature emerged, which supports the choice of linearity.

The experiments conducted on hills-and-valleys^25^ showed that mesenchymal cells tend to migrate toward the concave areas avoiding the convex and the saddle points; also, they tend to stabilize in the concave. The numerical model of Vassaux et al.^27^ shows that cell’s nucleus are mechanically relaxed in these concave areas. In Bade et al.^17^, fibroblasts favor concave rather than convex and flat by reorienting the lamellipodium along the concave contour. Cells were migrating parallel to the basal stress fibers when they usually migrate along apical stress fibers on flat^17^. On wavy sinusoidal substrates^24^, T-cells are surrounded by convex and they favor migration along concave valleys. This literature suggests that the lamellipodium has a role in maintaining the cell in the concave^24^, acting as a rudder which guides cells. In the case of a sinusoidal surface oriented along $\overrightarrow{y}$ (Eq. 1), the concave axis is given by $\pm \overrightarrow{x}$. More generally, we assume that cells follow the curvature contour given by the perpendicular vector to $\overrightarrow{\nabla }H$, denoted ${\overrightarrow{\nabla }}_{\perp }H(M)=\left(-{\partial }_{y}H\left(M\right),{\partial }_{x}H(M)\right)$.

If we denote $\theta$ the angle between ${\boldsymbol{v}}$ and ${\overrightarrow{\nabla }}_{\perp }H$, the realignment mechanism is,

6 $$\hat{\user2{w}}\left( {\kappa ,{\boldsymbol{p}}} \right) = \left\{ {\begin{array}{*{20}l} {\hat{\user2{p}}} \hfill & {\quad {\mathrm{if}}\user2{ }\kappa \left( {{\boldsymbol{r}}, \hat{\user2{p}}} \right) \le 0} \hfill \\ {\underline{{\underline {R} }} \left( {\alpha \theta } \right) \cdot \hat{\user2{p}}} \hfill & {\quad {\mathrm{if}}\user2{ }\kappa \left( {{\boldsymbol{r}}, \hat{\user2{p}}} \right) > 0} \hfill \\ \end{array} } \right.,$$

with $$\underline{{\underline {R} }} \left( {\alpha \theta } \right) = \left( {\begin{array}{*{20}l} {\cos \,\alpha \theta } \hfill & { - \sin \,\alpha \theta } \hfill \\ {\sin \,\alpha \theta } \hfill & {\cos \,\alpha \theta } \hfill \\ \end{array} } \right)$$ the rotation matrix and $\alpha$ a unitless constant in $[\mathrm{0,1}]$. In the context of cell migration in parallel ridges, a previous model used a similar realignment mechanism^50^. Finally, the cell position is obtained by integration of the velocity ${\boldsymbol{v}}=\frac{\mathrm{d}{\boldsymbol{r}}}{\mathrm{d}t}$.

### Parameters ${\Pi }_{\mu }$, ${S}_{\mu }$, $\gamma$ and $\alpha$

The model is driven by six parameters ${\Pi }_{\mu }$ and ${S}_{\mu }$ with $\mu \in \{x,y\}$ expressed in s and in µm/s respectively, $\gamma$ expressed in µm and $\alpha$ unitless. The two latter are free parameters but ${\Pi }_{\mu }$ and ${S}_{\mu }$ were extracted from experimental trajectory analysis and depend on the wavelength. For each experimental condition, the pool of trajectories is randomly divided in two subgroups. The first group is dedicated to parameters extraction as presented here; the second group is dedicated to comparisons with simulations (see Section “Results”).

For each trajectory, the MSD is computed in both $x$ and $y$-axis^45,51^,

7 $$MSD_{\mu } \left( {m{\Delta }t} \right) = \frac{1}{{N_{T} - m}} \mathop \sum \limits_{k = 0}^{{N_{T} - m - 1}} \left| {r_{\mu } \left( {\left( {k + m} \right){\Delta }t} \right) - r_{\mu } \left( {k{\Delta }t} \right)} \right|^{2} , \quad \mu \in \left\{ {x,y} \right\},$$

with $\Delta t$ the time step between two consecutive position measurement, ${N}_{T}$ the number of points in the trajectory and ${r}_{\mu }$ the cell position in the $\mu$-axis. The integer $m$ controls the time-lag window and goes from $0$ to ${N}_{T}-1$. When calculating the MSD, it is advised to avoid using the ${N}_{T}$ points in the trajectory. Since the MSD is an average, the number of available points decreases as the time lag increases, reducing the accuracy of the calculation and lowering the resolution at higher time lags. Moreover, MSD accuracy is affected by localization error and it does not necessarily depend on using all trajectory points to achieve precision^52,53^. We therefore calculated the MSD by considering the first third of the data points^45^.

The OU process exhibit a superdiffusive dynamic with a known theoretical MSD given by^42,45^,

8 $$MSD_{\mu }^{OU} \left( {\uptau } \right) = \left( {{\Pi }_{\mu } S_{\mu } } \right)^{2} \left( {e^{{ - \frac{{\uptau }}{{{\Pi }_{\mu } }}}} + \frac{{\uptau }}{{{\Pi }_{\mu } }} - 1} \right) + 2\sigma_{\mu }^{2} , \mu \in \left\{ {x,y} \right\},$$

with $\uptau$ the time lag and ${\sigma }_{\mu }$ the localization error. Performing a non-linear least square fit between Eqs. (7 and 8) enables to get ${\Pi }_{\mu }$ and ${S}_{\mu }$ values. To ensure that the results do not depend on a particular random draw, we repeated the process ten times with new random splits each time. For each condition, there are 60 trajectories which are split in two subgroups of 30 trajectories each (see Supplementary Information).

**Fig. 2:**
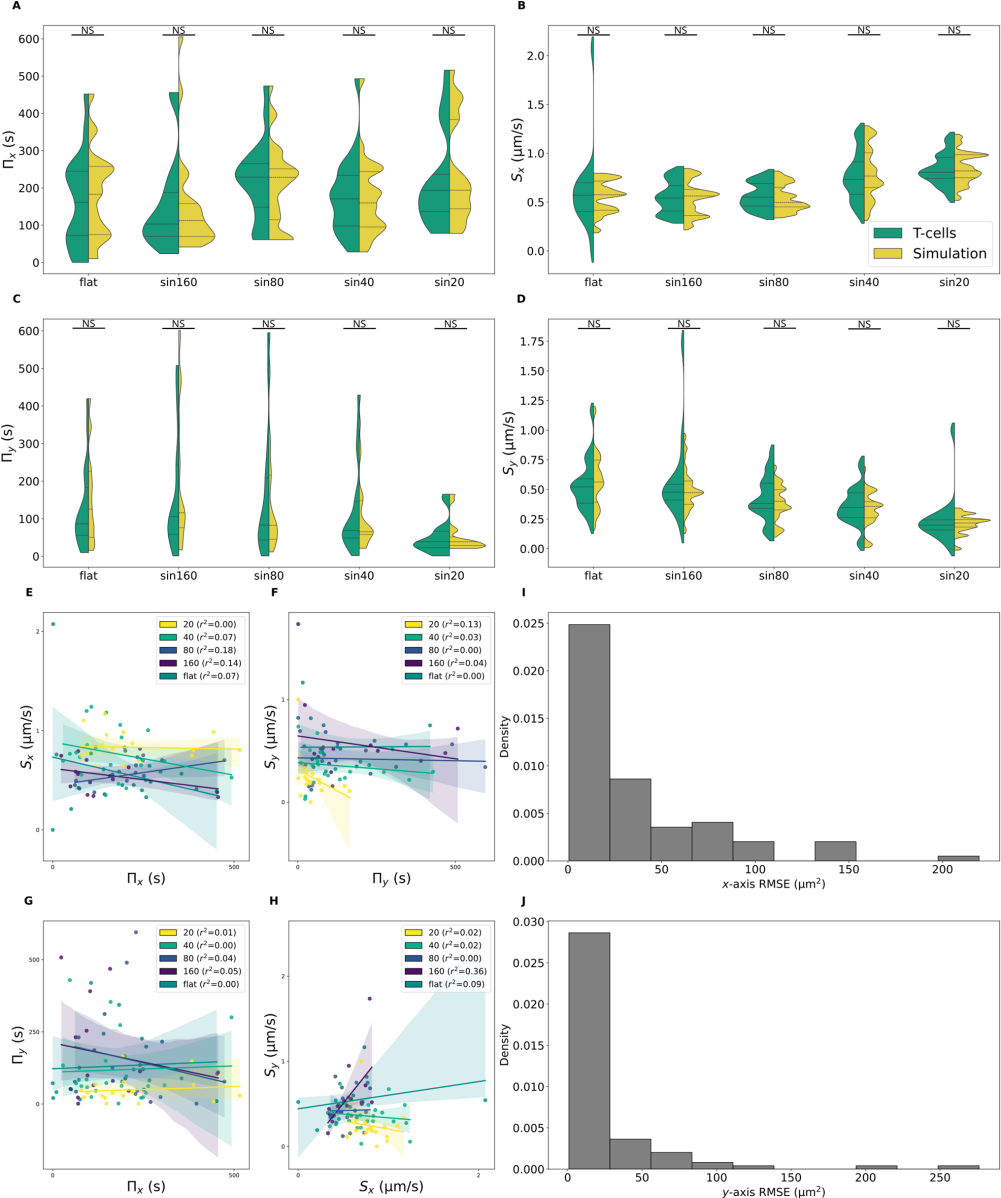
(**A**–**D**) ${\Pi }_{x}$, ${\Pi }_{y}$, ${S}_{x}$ and ${S}_{y}$ distributions either extracted from experimental trajectories of T-cells or generated from a probability density function (non-parametric Mann–Whitney U test, NS: not significant $p>0.05$). (**E**–**H**) The square of the Pearson correlation coefficient is computed to evaluate the correlation between parameters. (**I**–**J**) The RMSE between the experimental MSD and the fitted MSD is computed for each axis.

Figure 2 presents the results for a randomly selected split that is representative of the overall group. Figure 2e–h show that the parameters are independent either between the $x$ and $y$-axis and between $\Pi$ and $S$ meaning that we can decorrelate the distributions for assigning parameters values. For each fit, the Root Mean Squared Error (RMSE) was computed^46^ (Fig. 2i,j). The RMSE values ($\sim {10}^{2}$ µm^2^) are low compared the MSD order of magnitude ($\sim {10}^{4}$ µm^2^) meaning that the selected fits are good. Due to the exclusion of some cells (e.g. for a too short trajectory), each experimental distribution in Fig. 2 contains fewer than 30 tuples $\left({\Pi }_{x}, {\Pi }_{y}, {S}_{x}, {S}_{y}\right)$. If we assign these tuples to simulated cells, there would be fewer than 30 cells per simulation. However, we aim to have at least as many cells in the simulations as in the experiments, if not more. So, for each condition and each parameter, we generate a distribution with an equivalent probability density function ${\mathbb{P}}\left(X={x}_{i}\right)={p}_{i}$; then we draw as much tuples as needed. This process is performed with the OpenTURNS Python package. We draw 300 tuples from these distributions, Fig. 2a–d show parameters distributions both extracted and generated.

The parameters distributions are dependent to curvature. When curvature increases, the speed $S$ increases along $x$ and decreases along $y$. The persistence along $y$ decreases also with curvature. This suggests that the cell polarity and cell activity is more focused along the $x$-axis when curvature increases.

The values for the free parameters $\gamma$ and $\alpha$ are given in Table 1. We do not have information on how these parameters could be distributed in a cell population, so we choose a unique $\gamma$ and $\alpha$ values for the whole population.

**Table 1:** Parameters used for the simulations (N.A.: not applicable).

Simulation condition	$\gamma$ (µm)	$\alpha$
Flat	N.A	N.A
$\lambda =160$ μm	30	0
$\lambda =80$ μm	70	0.35
$\lambda =40$ μm	50	0.4
$\lambda =20$ μm	180	0.5

### Simulations and trajectory analysis

For each of the ten parameters sets generated in sec II.3, we run a simulation to check that the results are not overly influenced by a specific split. The simulated data are compared to the second subgroup not used to extract parameters. The simulated data consists of cell trajectories, representing consecutive positions over time. To characterize this data, the MSD and the velocity autocorrelation function (VACF) are widely used tools^41,42,45,54–56^. The MSD is a measurement of the cell diffusion. The MSD in a given axis was computed (Eq. 7), one can also compute the total MSD by summing $MS{D}_{x}$ and $MS{D}_{y}$ and average over the whole cell population. The VACF, denoted ${c}_{vv}$, indicates how velocity correlates in time, in a certain way it is a measurement of the cell persistence. For a single trajectory, ${c}_{vv}$ is given by^51^,

9 $$c_{vv} \left( {m{\Delta }t} \right) = \frac{1}{{N_{T} - m}} \mathop \sum \limits_{k = 0}^{{N_{T} - m - 1}} {\boldsymbol{v}}\left( {\left( {k + m} \right){\Delta }t} \right) \cdot {\boldsymbol{v}}\left( {k{\Delta }t} \right),$$

which is then averaged over the whole population. It is expected to get a ${c}_{vv}$ curve which decreases in time because of loss of memory for large-range time window, the question is how this curve decreases. For example, it is known for an OU process that the VACF presents an exponential collapse^45,57^. Time functions caracterize diffusion but cannot quantify the spatial bias observed in the resultant trajectories of cells. To do so, we introduce the area moment of inertia tensor, a $d\times d$ tensor, where $d$ is the spatial dimension where cells evolute; in our case $d=2$. This tensor describes the spatial distribution of trajectory points in the $xy$-plane and is useful to determine if there is a privilegied axis of migration caused by anisotropic surface^58,59^. The tensor components are given by ${I}_{\mu \nu }=\sum_{cells}\sum_{i=0}^{{N}_{T}-1}\Delta {r}_{\mu }\left({t}_{i}\right)\Delta {r}_{\nu }\left({t}_{i}\right)$, with $\Delta {r}_{\mu }\left({t}_{i}\right)={r}_{\mu }\left({t}_{i+1}\right)-{r}_{\mu }\left({t}_{i}\right),\mu ,\nu \in \{x,y\}$^60^. This tensor is symetric (${I}_{xy}={I}_{yx}$). Standard algebric method enables to get the two eigenvalues,

10 $$\lambda_{1,2} = \frac{1}{2}\left( {I_{xx} + I_{yy} \pm \sqrt {I_{xx}^{2} + I_{yy}^{2} - 2I_{xx} I_{yy} + 4I_{xy}^{2} } } \right).$$

**Fig. 3:**
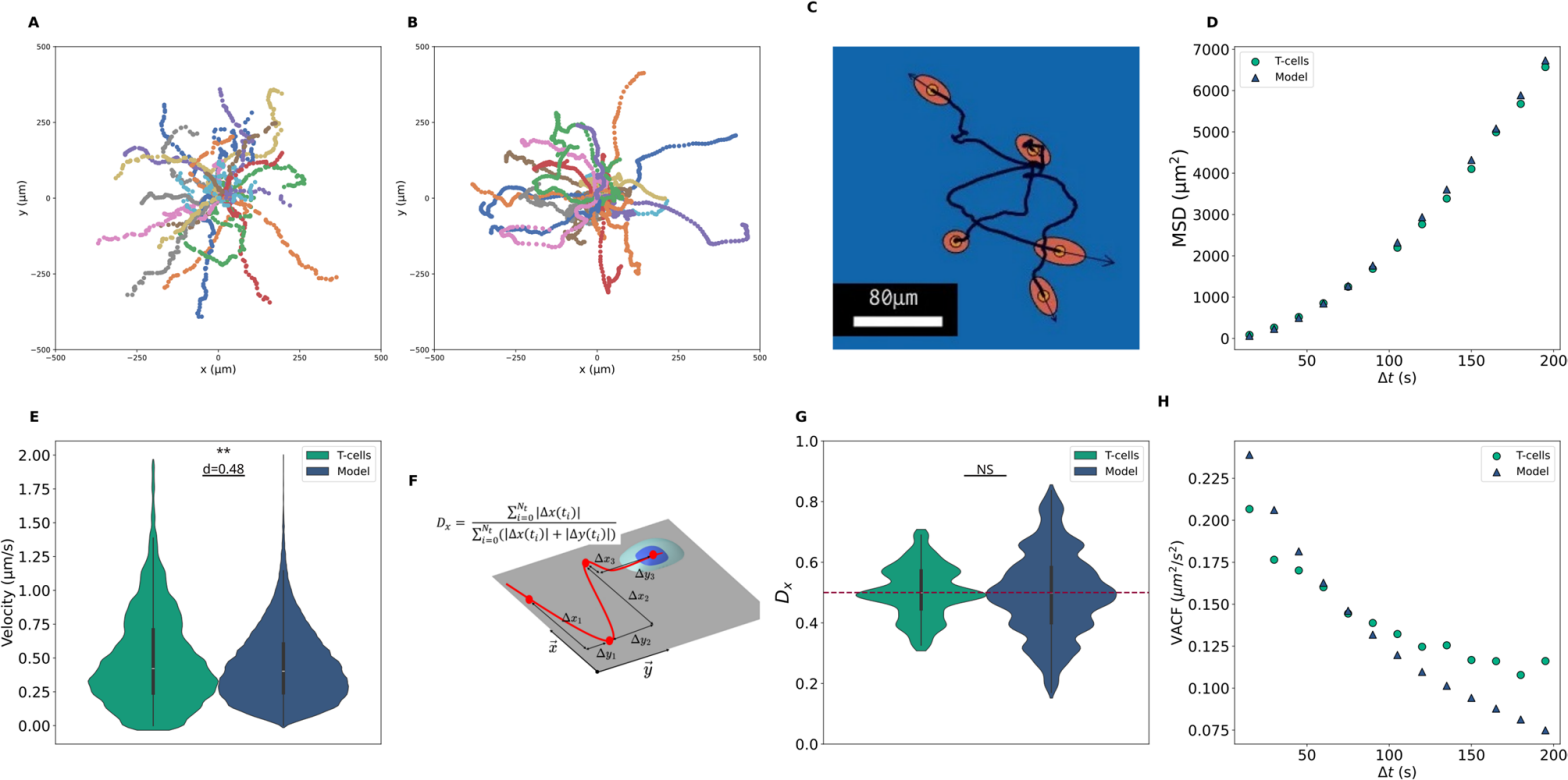
Results for a representative simulation among ten repetitions. (**A**) Experimental trajectories of T-cells over flat surface ($n=30$). (**B**) Trajectories obtained from simulation over flat surface. Simulation was conducted with 300 cells but for readability, 30 trajectories randomly chosen are shown. (**C**) Screenshot of the simulation after 7 min. The black lines are the trajectories, the black arrow are the instantaneous velocities. The cytoplasm and the nucleus are drawn but have no effect on the cell dynamic. For readability, only five cells are shown. (**D**) Experimental (green circles) and numerical (blue triangles) MSD curves, RMSE = 109 µm^2^. (**E**) Instantaneous velocity distribution $||{\boldsymbol{v}}||$. (**F**) Definition of the directional bias ${D}_{x}$ for a single trajectory and (**G**) ${D}_{x}$ distribution for experimental and simulated trajectories. Both distributions show no directional bias with a mean at 0.5. (**H**) T-cells and numerical VACF curves, RMSE = 0.05 µm^2^ s^-2^. (non-parametric Mann–Whitney U test, NS: not significant $p>0.05$, **: $p<0.01$).

If we choose the convention ${\lambda }_{1}\ge {\lambda }_{2}$, the ratio ${\lambda }_{1}/{\lambda }_{2}$ is equal to 1 in case of an isotropic flat surface and increases with the surface anisotropy. Finally we introduce a directional bias denoted ${D}_{x}$ defined in the experimental paper^24^ and in Fig. 3f. For one single trajectory,

11 $$D_{x} = \frac{{\mathop \sum \nolimits_{i = 0}^{{N_{t} - 1}} \left| {{\Delta }r_{x} \left( {t_{i} } \right)} \right|}}{{\mathop \sum \nolimits_{i = 0}^{{N_{t} - 1}} \left( {\left| {{\Delta }r_{x} \left( {t_{i} } \right)} \right| + \left| {{\Delta }r_{y} \left( {t_{i} } \right)} \right|} \right)}}.$$

This indicator provides the ratio of the distance traveled along the $x$-axis to the total distance. It takes a value of 0.5 when there is no directional bias, approaches 1 when the bias is along the $x$-axis, and approaches 0 when the bias is along the $y$-axis. It differs from the eigenvalues ratio which gives informations on the surface anisotropy through observed trajectories.

A convenient quantity to assess is cell instantaneous velocity given by,

12 $${\boldsymbol{v}}\left( {t_{i} } \right) = \frac{{{\boldsymbol{r}}\left( {t_{i + 1} } \right) - {\boldsymbol{r}}\left( {t_{i} } \right)}}{{{\Delta }t}}.$$

The distribution of $\Vert {\boldsymbol{v}}\Vert$ counts numerous values because of the large number of cells and trajectories points, either for the numerical or the experimental data. To statistically compare these non-normal distributions, we compute a Mann–Whitney U test. Because of the large ensemble size, a usual significance test is not enough to completely characterizing the data, one can add effect size analysis. The most popular is the Cohen’s d which requires at least normalized data^61^, instead here, we compute a non-parametric estimator for common-language (CL) effect size, denoted $d$, which does not require prerequisite assumption on the data^62^. In Eq. (12), $\Delta t$ is the sampling time step between two consecutives frames. The numerical $\Delta t$ is chosen to be the same as the experimental one.

## Results

### Flat surface

The first simulations are on a flat and homogeneous surface. In such a case, ${\boldsymbol{v}}={\boldsymbol{p}}$ and the model is an anisotropic and heterogeneous OU process (Fig. 3c). The model has simulated the migration of 300 cells during 20 min. The trajectories of modeled cells was sampled every 15 s. The integrating time step is chosen to be a hundred time smaller than the sampling time step^45^.

Figure 3 shows the quantities presented in Section “Simulations and trajectory analysis” computed for one representative simulation among the ten conducted. We found that the mean RMSE between the experimental and numerical curves for both the MSD and the VACF are respectively $410$ µm^2^ and $0.04$ µm^2^ s^−2^ with standard deviation $391$ µm^2^ and $0.01$ µm^2^ s^−2^. Both VACF curves are not totally following the same tendencies, the numerical curve decreases faster. The numerical ${D}_{x}$ factor mean is close to 0.5 which shows that the OU process has no preferential direction. We performed a nonparametric Mann–Whitney U test which gives no statistical difference between the two distributions ($p>0.05$) except for one among the ten. In this particular split, the trajectories subgroup dedicated for comparison has a biased ${D}_{x}$ median above $0.5$, leading to parameters distributions generated using trajectories with a biased ${D}_{x}$ median below $0.5$. In general, the numerical distribution ${D}_{x}$ is larger than the experimental one because the numerical population is larger with more outliers. Finally, we computed the instantaneous velocity distribution $\Vert {\boldsymbol{v}}\Vert$ (Fig. 3e). We performed a significance test which results in a statistical difference ($p<0.01$) at every simulation but mean size effect is $d=0.47$, i.e. there is a $47$% chance that a randomly selected numerical velocity is larger than a randomly selected T-cells velocity; the two distributions mostly overlap.

### Curved surface

**Fig. 4:**
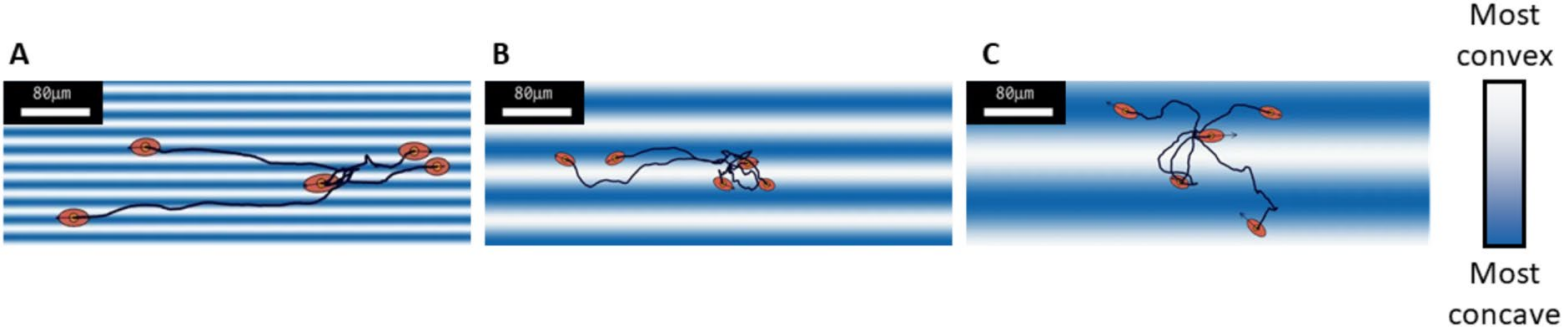
Simulation screenshots for $\lambda =20$ µm (**A**), $\lambda =$ 40 µm (**B**), $\lambda =160$ µm (**C**) all at $t\approx 7$ min. Black lines represent trajectories and black arrows are instantaneous velocities. Simulations are run with 300 cells but for readability, only five randomly chosen cells are shown. Blue is for concave valleys and white is for convex hills.

We reproduced numerically experiments on corrugate sinusoidal surfaces. The wavelength took values 20, 40, 80 and $160$ µm, amplitude was fixed at $a=10$ µm and model ran for 20 min with a sampling time of 15 s (Fig. 4). For each condition, we reproduce the simulations ten times with different split each time.

**Fig. 5:**
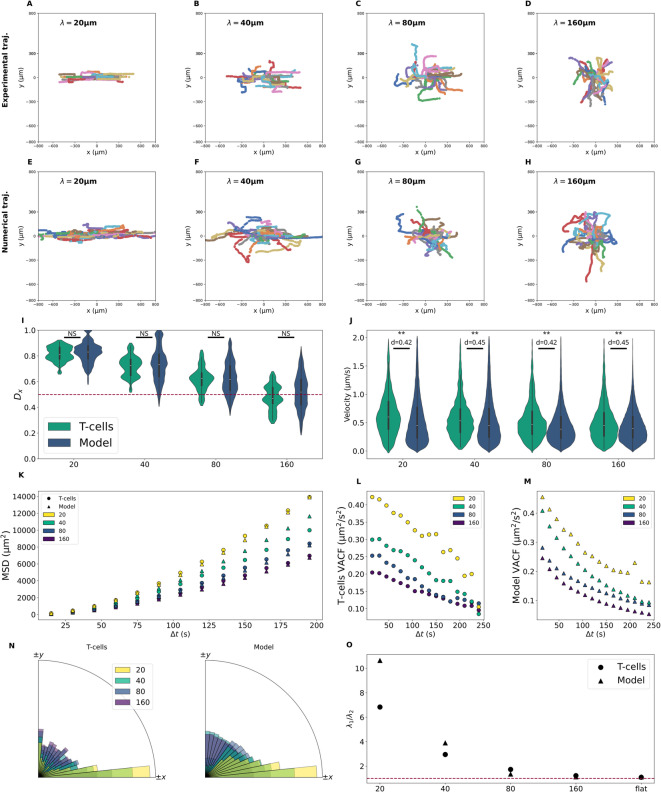
(**A**–**D**). Experimental trajectories of T-cells over sinusoidal surface with different wavelengths. $n=30$ for each experimental condition. (**E**–**H**). Trajectories obtained with simlulations. Simulations are run with 300 cells but for readability, only 30 randomly chosen cells are shown. (**I**) The directional bias ${D}_{x}$ distributions depends on the wavelength. The red dot line is ${D}_{x}=0.5$ when there is no bias. (**J**) Instantaneous velocity distribution depends on the wavelength. For readability, velocities above 2 µm/s have not been plotted, which represents 0.6% of the data. (**K**) The MSD increases with curvature meaning that curvature increases cell diffusion. RMSE are 212, 800, 229 and 207 µm^2^ for wavelength 20, 40, 80 and 160 µm respectively. (**L**) T-cells VACF (**L**) and numerical VACF (**M**) exhibit different behavior. RMSE between experimental and numerical curves are 0.06, 0.05, 0.02 and 0.04 µm^2^ s^−2^ for wavelength 20, 40, 80 and 160 µm respectively. (**N**) Instantaneous cells orientation. (**O**). Ratio of the eigenvalues of the quadratic moment of area tensor. The model captures the anisotropy of the bias for wavelength 40, 80 and 160 µm but overestimates this fraction for short wavelength (20 µm). (non-parametric Mann–Whitney U test, NS: not significant, **: $p<0.01$).

Figure 5 presents the comparison between experimental and numerical trajectories for a representative simulation chosen from the ten conducted. Simulated trajectories (Fig. 5e–h) are longer than experimental trajectories (Fig. 5a–d) because of the microscopy set-up limits. Experimental imaging lasted 20 min, nonetheless most of the cells were tracked for a shorter time because of track loss due to cell death or cell leaving the tracking field, which is not considered in the simulations. However simulation shows the same results as the experiments: the more the curvature increases (i.e. the wavelength decreases) the more the trajectories are aligned with the valleys. Figure 5i–o show quantitative analysis of numerical data systematically compared to experimental data. These analysis features were obtained from two types of data: the cell velocities (Fig. 5j, l–n) or the cell trajectories (Fig. 5i,k,o).

Figure 5n shows the bias in the cell direction of migration for both experimental and numerical velocities. T-cells are mostly migrating along the direction of the valley and this effect increases with curvature. For large wavelength ($160$ µm), although T-cells orientation distribution is mostly homogeneous there is still an alignement bias along the $x$-axis. There is also a bias along the $y$-axis which corresponds to when T-cells migrate from one valley to another and are facing the convex. This behavior is observable in experimental trajectories for short wavelengths where T-cells are migrating along a valley then doing a right-angle changing of direction to reach a neighboring valley (Fig. 5a,b).

The velocity is characterized also by its intensity (Fig. 5j). For each wavelength, the exprimental and numerical instantaneous velocity distributions are significatively different (Mann–Whitney U test, $p<0.01$) but the CL effect size is on average $d=0.44$ (Table 2); meaning again that both distributions are mostly overlapping each others for each condition.

**Table 2:** Mean and standard deviation (SD) of the RMSE and the effect size averaged over the ten simulations conducted.

Simulation condition	RMSE		Effect size
	MSD (µm^2^)	VACF (µm^2^ s^−2^)	$\Vert {\boldsymbol{v}}\Vert$
Flat (µm)	410 ± 391 (SD)	0.04 ± 0.01 (SD)	0.47 ± 0.03 (SD)
$\lambda =160$ μm	472 ± 313	0.04 ± 0.02	0.44 ± 0.03
$\lambda =80$ μm	418 ± 272	0.04 ± 0.02	0.43 ± 0.02
$\lambda =40$ μm	994 ± 504	0.05 ± 0.01	0.46 ± 0.03
$\lambda =20$ μm	1010 ± 429	0.07 ± 0.03	0.43 ± 0.03

In both experimental and numerical VACF (Fig. 5l–m), we observe that curvature enhances velocity correlation over short time periods. However, the decline patterns differ: while curvature does not influence the exponential decrease of the numerical VACF, the experimental VACF rapidly drops to zero. We attribut this abrupt collapse to the right-angle directional changes mentioned earlier, which disrupt velocity correlation. Still the model and experimental values have the same order of magnitude.

As a metric, the bias ${D}_{x}$ (Fig. 5i, defined in Fig. 3f) differs from the cell alignement (Fig. 5n) because it is computed from cells trajectories and not velocities. This indicator have been introduced in the experimental paper^24^ and captures the bias induced by curvature by increasing with curvature. In the case of $\lambda =160$ µm, we observed that for most of the split, ${D}_{x}$ mean is below $0.5$ when the whole population has a ${D}_{x}$ mean of $0.5$. Despite this difference, the ${D}_{x}$ mean of computed trajectories is always close to $0.5$.

The model captures this feature well, each numerical distribution have no statistical difference with its corresponding experimental distribution (non-parametric Mann–Whitney U test, $p>0.05$). This indicator suggests an anisotropic cell diffusion which is confirmed with the area moment of inertia tensor eigenvalues ratio ${\lambda }_{1}/{\lambda }_{2}$ (Fig. 5o). The model follows the experimental tendency except for the shortest wavelength $20$ µm, where the model surestimates the anisotropy (relative error is 56%). The change in anisotropy supports the assumption of decomposing the cell dynamic in two directions (Eq. 3).

Lastly, the MSD is computed (Fig. 5k) to assess cell diffusion in space. When curvature increases the cell diffusion is much more efficient and follows a superdiffusive dynamics as an OU process. The MSD is almost multiplied by two on highest curved surface ($\lambda =20$ µm) compared to flat surface at $\Delta t=200$ s. For each of the ten simulations, the RMSE for the MSD has been computed and averaged (Table 2). The standard-deviation is high meaning that the MSD curve depends on the split. However, the RMSE ($\sim {10}^{3}$) is one order of magnitude below the MSD order of magnitude ($\sim {10}^{4}$). While this result is not highly precise, they remain within an acceptable range.

### The role of $\Pi$ and $S$

To understand better the impact of curvature on persistence, simulations are conducted but $\Pi$ and $S$ parameters distributions are switched. We focus on curved surface with $\lambda =20$ µm because this is the condition where the curvature effets are the more important. We conducted four simulations:

- ${\Pi }^{F}, {S}^{C}$: the persistance distribution for flat surface (F) is used, the speed distribution is untouched (C).
- ${\Pi }^{C}, {S}^{F}$: the persistance distribution is untouched, the speed distribution for flat surface is used.
- ${\Pi }_{x}\leftrightarrow {\Pi }_{y}$: both $\Pi$ and $S$ distributions for curvature are used ($\lambda =20$ µm), the $x$ and $y$-axis $\Pi$ distributions are exchanged.
- ${S}_{x}\leftrightarrow {S}_{y}$: both $\Pi$ and $S$ distributions for curvature are used, the $x$ and $y$-axis $S$ distributions are exchanged.

**Fig. 6:**
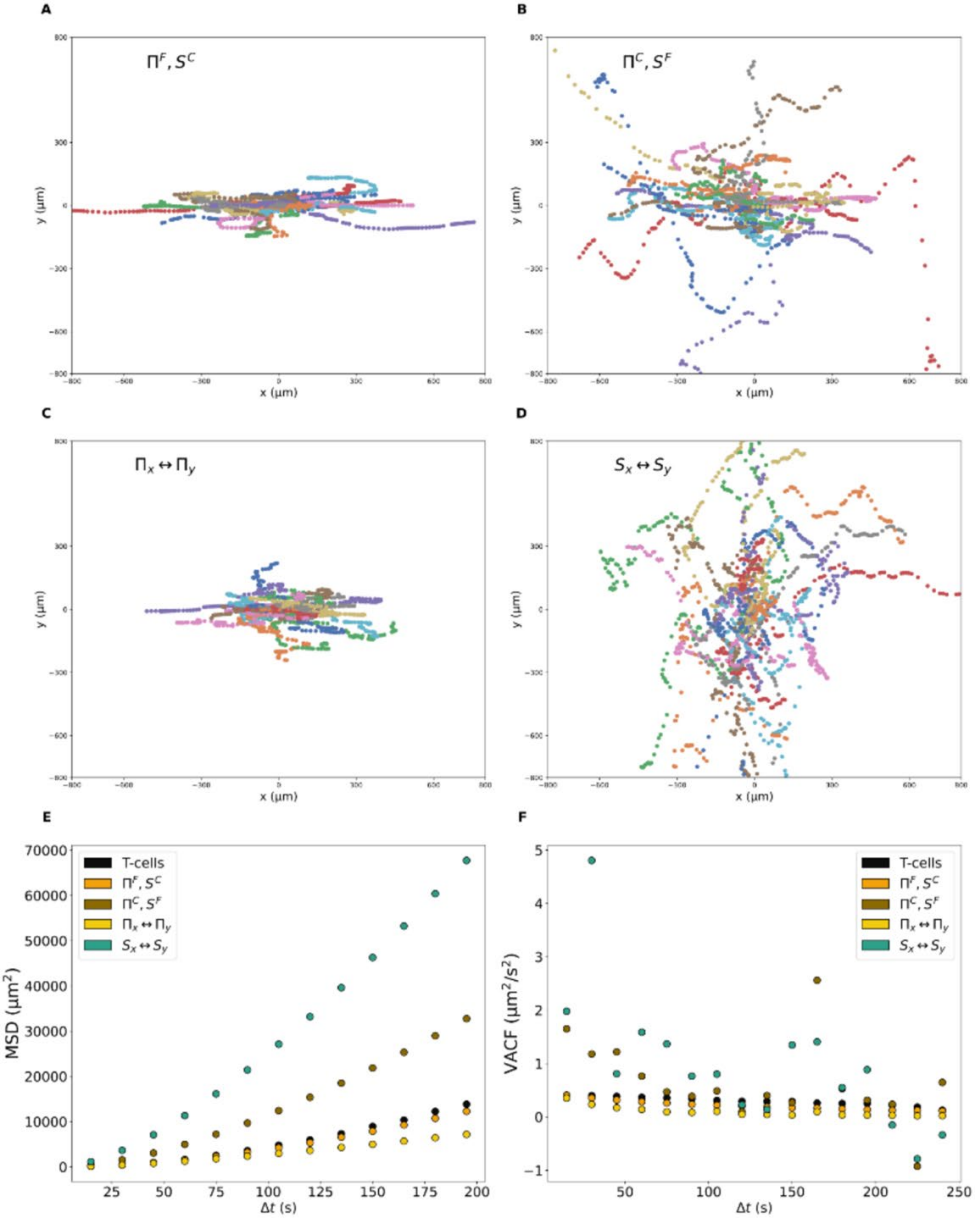
Simulation over curved surface ($\lambda =20$ µm). Same as before, the concave valleys are along the $x$-axis. $\Pi$ distribution is substituted with the distribution obtained under flat surface (**A**), and $S$ distribution with that from flat (**B**). Distributions are exchanged depending on the axis for $\Pi$ (**C**) or $S$ (**D**). MSD and VACF are computed (**E**–**F**).

MSD and VACF are metrics which depend on the persistence so they are computed. The important parameter seems to be $S$, when the speed distributions are changed, the MSD diverges and the VACF becomes noisy (Fig. 6). Surprisingly, using the flat persistence distribution (${\Pi }^{F}, {S}^{C}$) does not change quite the results and exchanging the persistance axis (${\Pi }_{x}\leftrightarrow {\Pi }_{y}$) reduces diffusion. In the two latter conditions, migration along the grooves axis is still favorated, the ${D}_{x}$ mean is close to 0.8 (data not shown) which is coherent with ${D}_{x}$ experimental distribution.

The curved $S$ distribution has a mean of $0.85$ µm/s in the $x$-axis and $0.21$ µm/s in the $y$-axis (Fig. 2b,d), thereby switching $x$ and $y$-axis (${S}_{x}\leftrightarrow {S}_{y}$) is like increasing the noise along the $y$-axis while reducing it along the $x$-axis, it promotes the transition from grooves to another. This is why cells are more likely to migrate along the $y$-axis (Fig. 6d); the ${D}_{x}$ mean is $0.3$ (data not shown). The cell ability to maintain a cap in valleys is enabled by an increase in noise (modulated by $S$) along the direction of valleys and a reduction of noise in the perpendicular axis. This promotes the migration in the concave valleys. When performing numerical simulations, it is essential that each parameter distribution be associated with the corresponding experimental condition. This suggests that the balance between persistence and noise is required for curvotaxis.

## Discussion

It appears that the parameter which modulates the noise amplitude, the speed $S$, plays a key role in the model validity. The noise amplitude is more important in the direction of the valley. The noise impacts the persistence inertia by random perturbations, it seems that persistence along the concave valleys can be maintained by an anisotropic noise which aligns along the cell direction of migration. It can translates to FA strength which are more tensed and stable in concave areas as reported for mesenchymal cells^25^. If FA tend to more stable in the valley axis, cells would promote the direction towards the valley.

The model parameters are derived from experimental trajectories, and the simulated trajectories are subsequently compared with experimental observations. To prevent circularity and overfitting, the experimental dataset is randomly split into two subgroups: one for parameter generation and the other for comparison with numerical results. For each condition, there are 60 trajectories. Dividing the data pool leads to a very few numbers of trajectories to work with. However, the model generates trajectories which present similar statistics to experimental one. Despite the limited amount of experimental data, the results present a fair level of accuracy. A larger dataset would have certainly resulted in distributions that are more representative of the T-cells population.

Several studies have highlighted the limitations of the OU process, which fails to fully reproduce the VACF curve, even on flat surfaces^45,54,57^. Two solutions are proposed in the literature: adopting a more complex model formulation^54,57^ or incorporating cellular heterogeneity into the OU process^45^. Including cellular heterogeneity is particularly relevant, as it allows for the avoidance of assuming average parameters across the population. Moreover, the fitting we have performed remains straightforward with the OU process.

Because the randomness observed experimentally has not been simulated by existing curvotaxis models which essentially consider the deterministic curvature factor on cell migration, we developed in this study a new curvotaxis model based on a stochastic approach through a PRW formulation based on an OU process. This very simple approach enables to model a cell population where each single cell has its own behavior, modeled by a stochastic term in the cell dynamic, and still a deterministic memory term for curvature action. The model includes cell heterogeneity among the population; each simulated single cell was attributed its own parameters values. We adapted the protocol in Wu and al.^45^; in our work we constructed probability density function from the parameters extracted distributions which enables to draw as much parameters required to run the simulation and not be limited by the number of tracked trajectories.

When the curvature is high, for short wavelengths, T-cells are preferentially migrating along the concave valleys then cross convex hills by turning perpendicularly. This mechanism is observable qualitatively on Fig. 5a,b and quantitatively with T-cells aligning along the valleys and also in the perpendicular direction (Fig. 5n). It is also visible with the VACF vanishing indicating the decorrelation of the velocities with time (Fig. 5l). Simulated cells migrate mostly along the concave valleys, the ${D}_{x}$ distributions indicate the same bias for T-cells and numerical trajectories, they are also able to cross convex hills. The VACF curves on flat surface are quite similar experimentally and numerically, but unlike T-cells, simulated cells do not exhibit a drop in the VACF curves on curvature. This suggests that the model does not entirely capture the action of curvature on persistence. We found that T-cells migration across curved landscape displays superdiffusive dynamics, with the MSD curve showing a characteristic increase similar to that of an OU process.

Our model is based on a single cell type, T-cells; however, other cell lines, such as mesenchymal cells, fibroblasts and fish keratocytes, are also known to be responsive to surface curvature^25,63^. It would be valuable to evaluate the model on these additional cell lines and different types of surfaces. This model presents a versatile paradigm which could be adapted to dynamic curvotaxis and multicell simulations.

## Supplementary Information

Supplementary Information 1.

Supplementary Information 2.

Supplementary Information 3.

Supplementary Information 4.

Supplementary Information 5.

Supplementary Information 6.

Supplementary Information 7.

Supplementary Information 8.

Supplementary Information 9.

Supplementary Information 10.

Supplementary Information 11.

## Data Availability

Experimental data has been provided by Kwang Hoon Song and Junsang Doh (Song et al. 2015). Model is available at the following github repository https://github.com/GildasCarlin/anisotropic-PRW-model-for-curvotaxis.git.
