# Supplementary material for: Anisotropic persistent random walk model simulates T-cells migration over curved landscapes
Source: Sci Rep. 2025 Jun 4;15:19629. doi: 10.1038/s41598-025-02804-3 (PMC12137650; doi:10.1038/s41598-025-02804-3)
Supplement: Supplementary file 1 — Supplementary Information 1. [file 41598_2025_2804_MOESM1_ESM.pdf]

Anisotropic persistent random walk model simulates T-cells migration over curved landscapes

Gildas Carlin[1,2,4*], Ian Manifacier[1,2,4*], Dang Khoa Cao[3], Laurent Pieuchot[3], Valeriy Luchnikov[3], Jean-Louis Milan[1,4,*]

1. Aix Marseille Univ, CNRS, ISM, Marseille, France

2. Aix Marseille Univ, APHM, CNRS, ISM, Sainte-Marguerite Hospital, Institute for Locomotion, Department of Orthopaedics and Traumatology, Marseille, France

3. Institut de Science des Matériaux de Mulhouse (IS2M), CNRS, Université de Haute-Alsace, UMR 7361, 68100 Mulhouse, France

4. Aix Marseille Univ, Gustave Eiffel Univ, LBA, Marseille, France

This is an open access article distributed under a Creative Commons Attribution 4.0 International (https://creativecommons.org/licenses/by/4.0/).

Supplementary Information

1. Supplementary movies

Movie S1 to S10 are videos of the simulations. The cytoplasm and the nucleus are drawn but have no influence on the cell dynamics. Blue areas correspond to concave, white areas correspond to convex.

Movie S1 Wavy sinusoidal surface with $\lambda = 20\mu\text{m}$, $n = 300$ cells.
Movie S2 5 cells randomly chosen migrating over a wavy sinusoidal surface with $\lambda = 20\mu\text{m}$.
Movie S3 Wavy sinusoidal surface with $\lambda = 40\mu\text{m}$, $n = 300$ cells.
Movie S4 5 cells randomly chosen migrating over a wavy sinusoidal surface with $\lambda = 40\mu\text{m}$.
Movie S5 Wavy sinusoidal surface with $\lambda = 80\mu\text{m}$, $n = 300$ cells.
Movie S6 5 cells randomly chosen migrating over a wavy sinusoidal surface with $\lambda = 80\mu\text{m}$.
Movie S7 Wavy sinusoidal surface with $\lambda = 160\mu\text{m}$, $n = 300$ cells.
Movie S8 5 cells randomly chosen migrating over a wavy sinusoidal surface with $\lambda = 160\mu\text{m}$.
Movie S9 Flat surface, $n = 300$ cells.
Movie S10 5 cells randomly chosen migrating over a flat surface.

2. More details on the process of generating the parameters

A nonlinear least-squares fit is performed between the theoretical expression of the MSD and the experimental MSD computed from a trajectory (Eq. 7 and 8 in the main text). We include localization error σ_μ because the fit is performed with an experimental MSD, which is subject to measurement errors. The residuals to minimize are given by,

$$R_\mu = MSD_\mu^{OU} - MSD_\mu^{exp}. \quad (1)$$

The MSD resolution decays when the time-lag τ increases, so the points for large τ are less reliable. Therefor we give less weight to the points calculated with a large time-lag. The square of the residuals is multiplied by a weight function,

$$W_{\mu,n} = \frac{1}{MSD_\mu^{OU}(n\Delta t)} \sqrt{\frac{-n^3 + 4Kn^2 + n + 2K}{6K^2 n}}. \quad (2)$$

The square root expression is the MSD relative error at $\tau = n\Delta t$ averaged with $K = N_t - n + 1$ measurements[1,2]. Fig. 1.a shows a fit of a trajectory on flat surface. Fig. 1.b is the distribution of the σ_μ for flat surface. These distributions are centered around 0 which indicates no bias in the localization error.

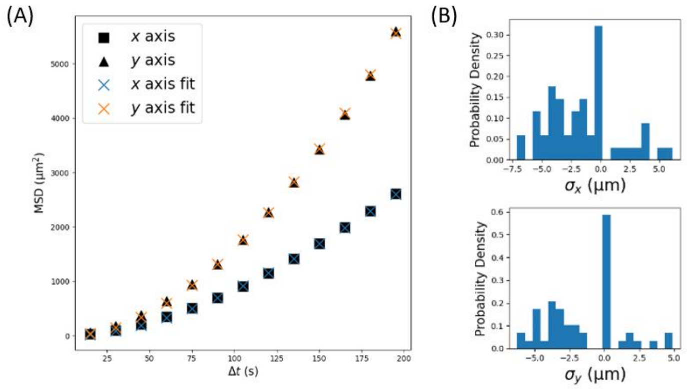

Figure 1: (A) Fit between experimental MSD and theoretical MSD for a representative trajectory on flat surface. (B) σ_μ distribution after fitting all trajectories on flat surface.

3. Subdivision of the experimental dataset

The experimental dataset is randomly divided in two subgroups. One of them is assigned to parameters generation, the remaining subgroup is assigned to model validation. We discuss here the number of cells required to get representative distributions either for the process of generating parameters or the comparison with simulated trajectory. We focus here on flat surface but these results are extendable to dataset on curvature.

The MSD is computed with random subgroups of 10, 20 or 30 trajectories among the 60 trajectories. The process is repeated multiple time, Fig. 2.a shows two representative draws. To have a representative MSD of the whole cell population which does not depend on a specific split, it requires at least 20 or 30 cells.

In the same line, (Π_μ, S_μ) distributions are generated using 10, 20 or 30 cells following the process described in Section II.3 in the main text (Fig. 2.b). A Mann-Whitney U test is performed between the generated distributions and the (Π_μ, S_μ) distribution generated using the whole dataset. The process is repeated multiple times, the number of times where there is a significant statistical difference ($p<0.05$) is counted. When 10 cells are used, the distributions are statistically significantly different 77% of the time. This percentage drops to 3% and 1.3% when 20 or 30 cells are drawn. So, we assume that 20 or 30 cells are required to get representative parameters distributions.

Finally, we choose to split the experimental dataset in half: a subgroup of 30 trajectories are assigned to parameters generation, the remaining 30 trajectories are assigned to model validation.

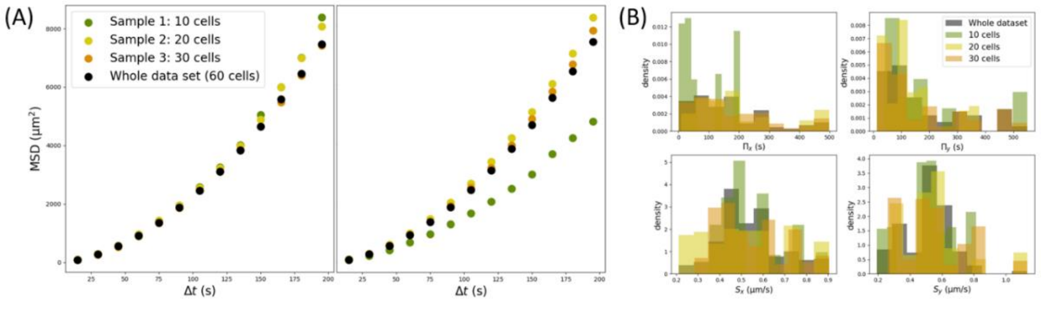

Figure 2: (A-B) T-cells MSD calculated with 10, 20 or 30 cells randomly chosen among 60 cells. Two representative draws are shown (B) Distributions are generated using data extracted from T-cells, using either 10, 20 or 30 cells.

4. Mean curvature and gradient of mean curvature

Following discussion is presenting standard differential geometry results[3]. Let M be a point on a surface, expressed in an orthogonal basis $(O, \vec{x}, \vec{y}, \vec{z})$,

$$\overrightarrow{OM} = \begin{pmatrix} x \\ y \\ h(x,y) \end{pmatrix}, \tag{3}$$

where h is at least a three-times differentiable function. The tangent vectors to the surface at M are expressed,

$$\vec{e}_x = \partial_x \overrightarrow{OM} = \begin{pmatrix} 1 \\ 0 \\ \partial_x h \end{pmatrix}, \quad \vec{e}_y = \partial_y \overrightarrow{OM} = \begin{pmatrix} 0 \\ 1 \\ \partial_y h \end{pmatrix}. \tag{4}$$

The metric tensor $\underline{\underline{g}}$ components are $g_{\mu\nu}(M) = \vec{e}_\mu(M) \cdot \vec{e}_\nu(M)$ with $\mu, \nu \in \{x, y\}$ which leads to,

$$\underline{\underline{g}}(M) = \begin{pmatrix} 1 + (\partial_x h)^2 & \partial_x h \partial_y h \\ \partial_x h \partial_y h & 1 + \left(\partial_y h\right)^2 \end{pmatrix}. \tag{5}$$

Denoting $g = \det \underline{\underline{g}} = 1 + (\partial_x h)^2 + \left(\partial_y h\right)^2$, the unit-length normal vector at point M is,

$$\vec{e}_z = \frac{\vec{e}_x \wedge \vec{e}_y}{\left\| \vec{e}_x \wedge \vec{e}_y \right\|} = \frac{1}{\sqrt{g}} \begin{pmatrix} -\partial_x h \\ -\partial_y h \\ 1 \end{pmatrix}. \tag{6}$$

The curvature tensor $\underline{\underline{K}}$ components are $K_{\mu\nu}(M) = \vec{e}_z(M)^T \cdot \partial_\mu \vec{e}_\nu(M)$ with $\mu, \nu \in \{x, y\}$ which is,

$$\underline{\underline{K}}(M) = \frac{1}{\sqrt{g}} \begin{pmatrix} \partial^2_{xx} h & \partial^2_{xy} h \\ \partial^2_{xy} h & \partial^2_{yy} h \end{pmatrix}. \tag{7}$$

With the definition of mean curvature H (eq. 2 main text), it is expressed only with h and its derivatives,

$$H(M) = \frac{g_{yy} \partial^2_{xx} h + g_{xx} \partial^2_{yy} h - 2 g_{xy} \partial^2_{xy} h}{2 g^{3/2}}. \tag{8}$$

If we denote $u(M) = g_{yy} \partial^2_{xx} h + g_{xx} \partial^2_{yy} h - 2 g_{xy} \partial^2_{xy} h$ and $v(M) = 2 g^{3/2}$, the gradient of mean curvature $\vec{\nabla} H$ reads,

$$\vec{\nabla} H = \left(\frac{v \partial_x u - u \partial_x v}{4 g^3}, \frac{v \partial_y u - u \partial_y v}{4 g^3} \right). \tag{9}$$

The partial derivatives of u and v are,

$$\begin{aligned}
\partial_x u &= g_{yy} \partial^3_{xxx} h + g_{xx} \partial^3_{xyy} h + 2 \partial^2_{xx} h \left(\partial^2_{xy} h \partial_y h + \partial^2_{yy} h \partial_x h \right) - 2 \left(g_{xy} \partial^3_{xxy} h + \partial^2_{xy} h \left(\partial^2_{xx} h \partial_y h + \partial^2_{xy} h \partial_x h \right) \right), \\
\partial_y u &= g_{yy} \partial^3_{xxy} h + g_{xx} \partial^3_{yyy} h + 2 \partial^2_{yy} h \left(\partial^2_{xx} h \partial_y h + \partial^2_{xy} h \partial_x h \right) - 2 \left(g_{xy} \partial^3_{xyy} h + \partial^2_{xy} h \left(\partial^2_{xy} h \partial_y h + \partial^2_{yy} h \partial_x h \right) \right), \\
\partial_x v &= 6 \left(\partial^2_{xx} h \partial_x h + \partial^2_{xy} h \partial_y h \right) g^{1/2}, \\
\partial_y v &= 6 \left(\partial^2_{xy} h \partial_x h + \partial^2_{yy} h \partial_y h \right) g^{1/2}.
\end{aligned} \tag{10}$$

5. Discretization of the model

The system of equation is,

$$\begin{cases} \dfrac{\mathrm{d}p_\mu}{\mathrm{d}t} = -\dfrac{p_\mu}{\Pi_\mu} + \sqrt{\dfrac{S_\mu^2}{\Pi_\mu}}\eta_\mu(t), \ \ \mu \in \{x, y\} \\ \|\boldsymbol{v}\| = f(\kappa, \boldsymbol{p}) \\ \theta(\boldsymbol{v}) = \widehat{\boldsymbol{w}}(\kappa, \boldsymbol{p}) \\ \dfrac{\mathrm{d}\boldsymbol{r}}{dt} = \boldsymbol{v} \end{cases} \tag{11}$$

With $\boldsymbol{p} = (p_x, p_y)$ the polarity vector, $\boldsymbol{v}$ the velocity vector, $\boldsymbol{r}$ the cell position and $\theta(\boldsymbol{u})$ the argument of the vector $\boldsymbol{u}$. Π_μ and S_μ are the OU process parameters. The randomness is modelled with a Wiener process η uncorrelated in time and a mean centered in 0,

$$\langle \eta(t) \rangle = 0, \qquad \langle \eta(t_1)\eta(t_2) \rangle = \delta(t_1 - t_2).$$

We discretize the system with a semi-explicit Euler scheme,

$$\begin{cases} p_\mu^{i+1} = \left(1 - \dfrac{\Delta t}{\Pi_\mu}\right) p_\mu^i + \sqrt{\dfrac{S_\mu^2 \Delta t}{\Pi_\mu}} n_\mu^i, \ \ \mu \in \{x, y\} \\ \|\boldsymbol{v}_i\| = f(\kappa_i, \boldsymbol{p}_i) \\ \theta(\boldsymbol{v}_i) = \widehat{\boldsymbol{w}}(\kappa_i, \boldsymbol{p}_i) \\ \boldsymbol{r}_{i+1} = \boldsymbol{r}_i + \boldsymbol{v}_i \Delta t \end{cases} \tag{12}$$

The integrating time-step Δt is chosen to be a hundred time smaller than the sampling time-step of 15s. The random number n_μ^i is generated with a Box-Muller transform[4] which is equivalent to drawing a number from a normal law $\aleph(0,1)$. To scale the random effect to the time step, we then multiply n_μ^i to a standard-deviation $\sqrt{\Delta t}$ [1,5,6].

References

1. Wu, P.-H., Giri, A., Sun, S. X. & Wirtz, D. Three-dimensional cell migration does not follow a random walk. *Proceedings of the National Academy of Sciences* **111**, 3949–3954 (2014).
2. Qian, H., Sheetz, M. P. & Elson, E. L. Single particle tracking. Analysis of diffusion and flow in two-dimensional systems. *Biophysical Journal* **60**, 910–921 (1991).
3. Kreyszig, E. *Differential Geometry By Erwin Kreyszig*. (1991).
4. Scott, D. W. Box–Muller transformation. *WIREs Computational Statistics* **3**, 177–179 (2011).
5. Wright, D. The digital simulation of stochastic differential equations. *IEEE Transactions on Automatic Control* **19**, 75–76 (1974).
6. Stokes, C. L., Lauffenburger, D. A. & Williams, S. K. Migration of individual microvessel endothelial cells: stochastic model and parameter measurement. *Journal of Cell Science* **99**, 419–430 (1991).
