# Supplementary figures and images for: Anisotropic persistent random walk model simulates T-cells migration over curved landscapes
Source: Sci Rep. 2025 Jun 4;15:19629. doi: 10.1038/s41598-025-02804-3 (PMC12137650; doi:10.1038/s41598-025-02804-3)

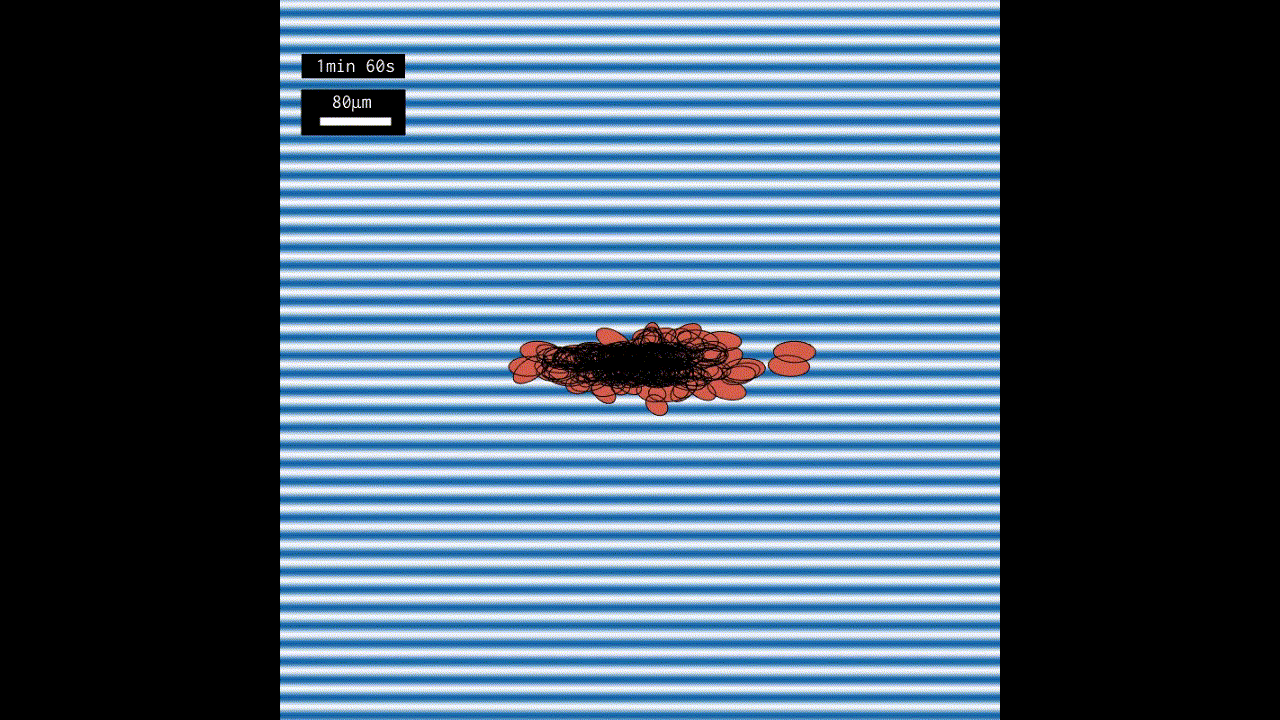

Supplement: Supplementary file 2 — Supplementary Information 2. [file 41598_2025_2804_MOESM2_ESM.gif]

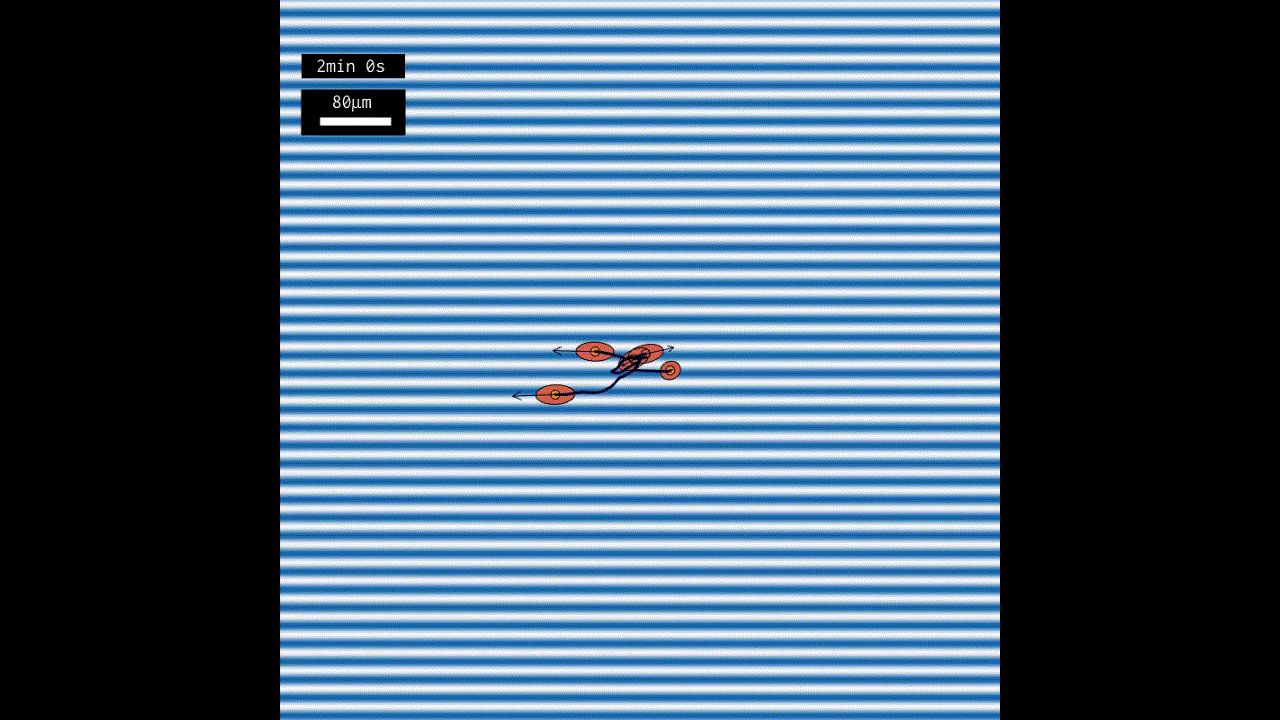

Supplement: Supplementary file 3 — Supplementary Information 3. [file 41598_2025_2804_MOESM3_ESM.gif]

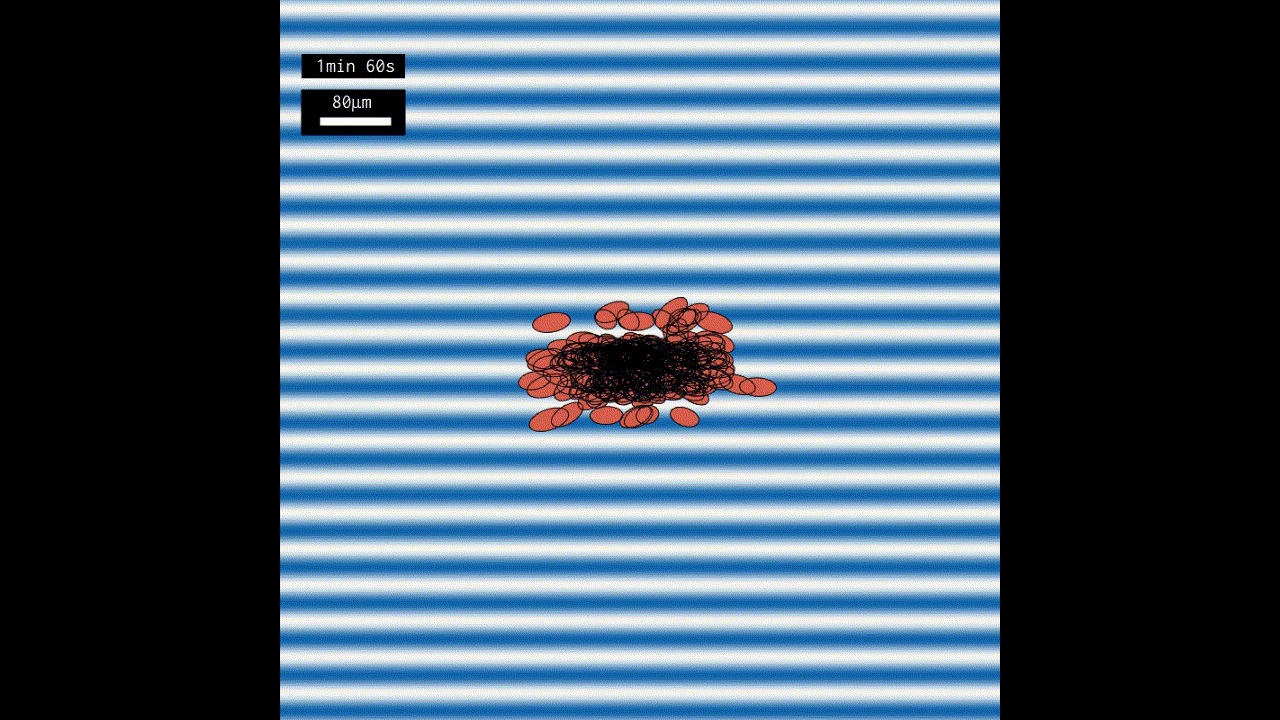

Supplement: Supplementary file 4 — Supplementary Information 4. [file 41598_2025_2804_MOESM4_ESM.gif]

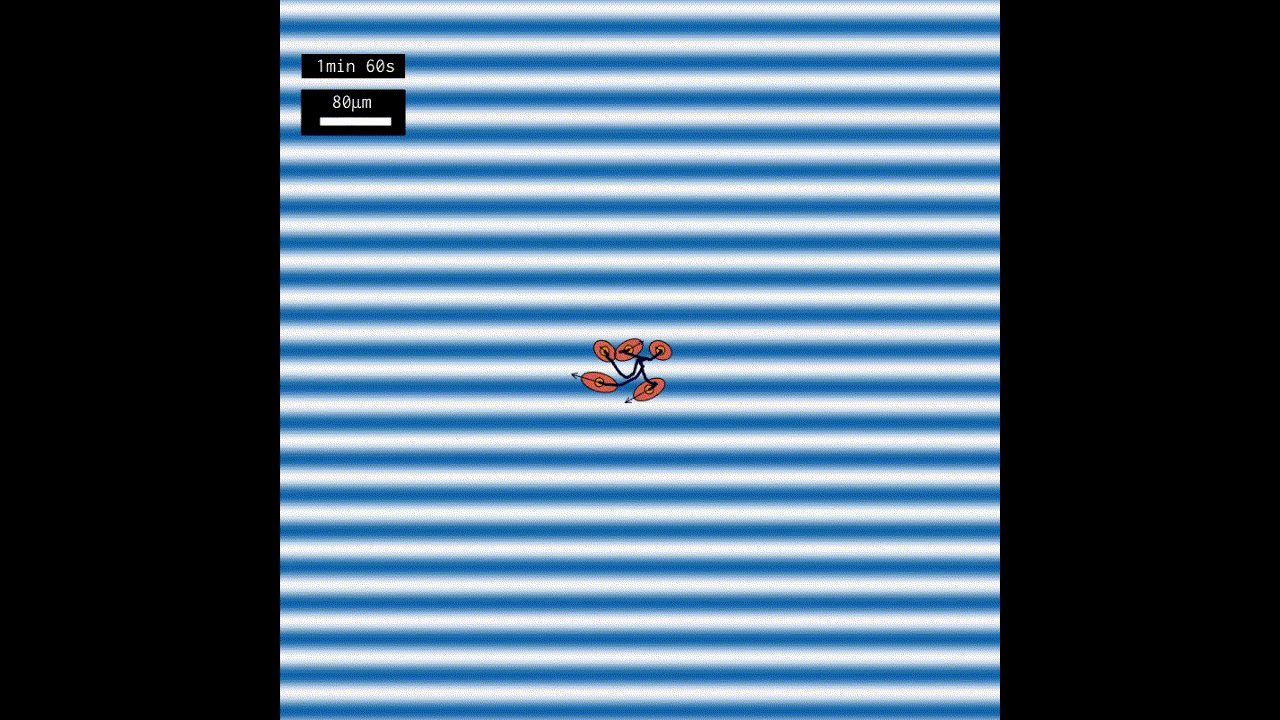

Supplement: Supplementary file 5 — Supplementary Information 5. [file 41598_2025_2804_MOESM5_ESM.gif]

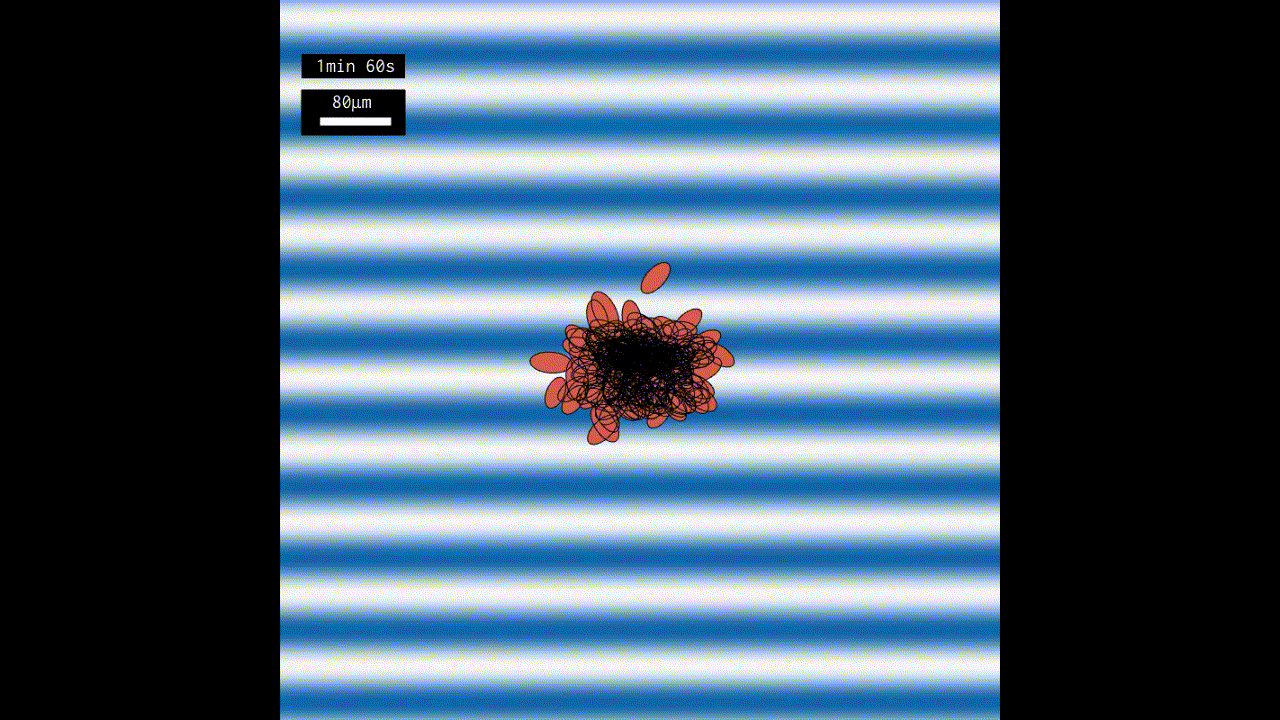

Supplement: Supplementary file 6 — Supplementary Information 6. [file 41598_2025_2804_MOESM6_ESM.gif]

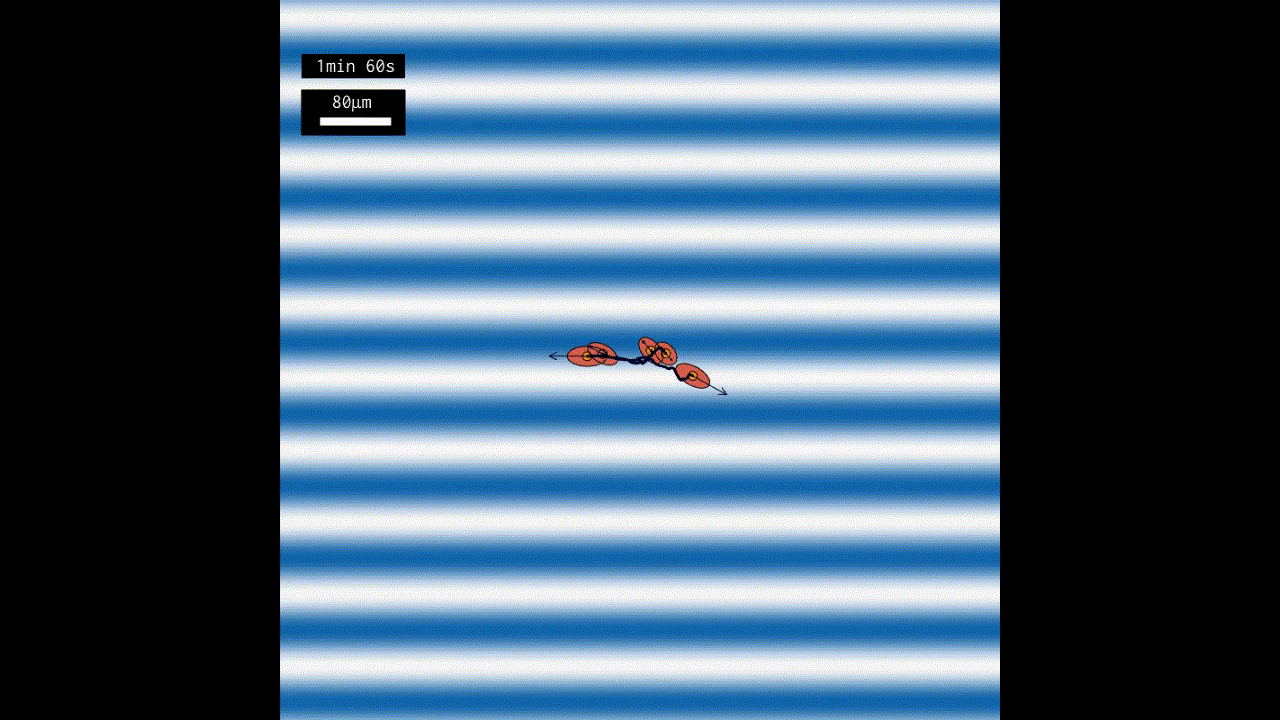

Supplement: Supplementary file 7 — Supplementary Information 7. [file 41598_2025_2804_MOESM7_ESM.gif]

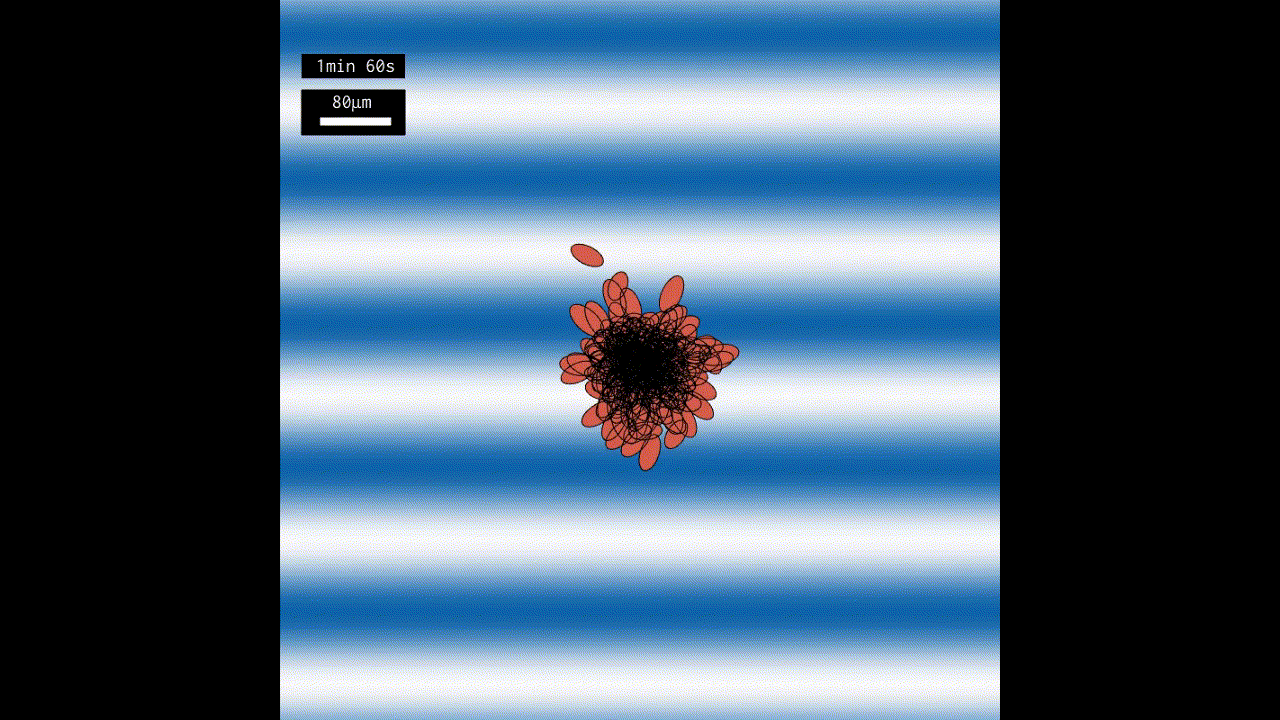

Supplement: Supplementary file 8 — Supplementary Information 8. [file 41598_2025_2804_MOESM8_ESM.gif]

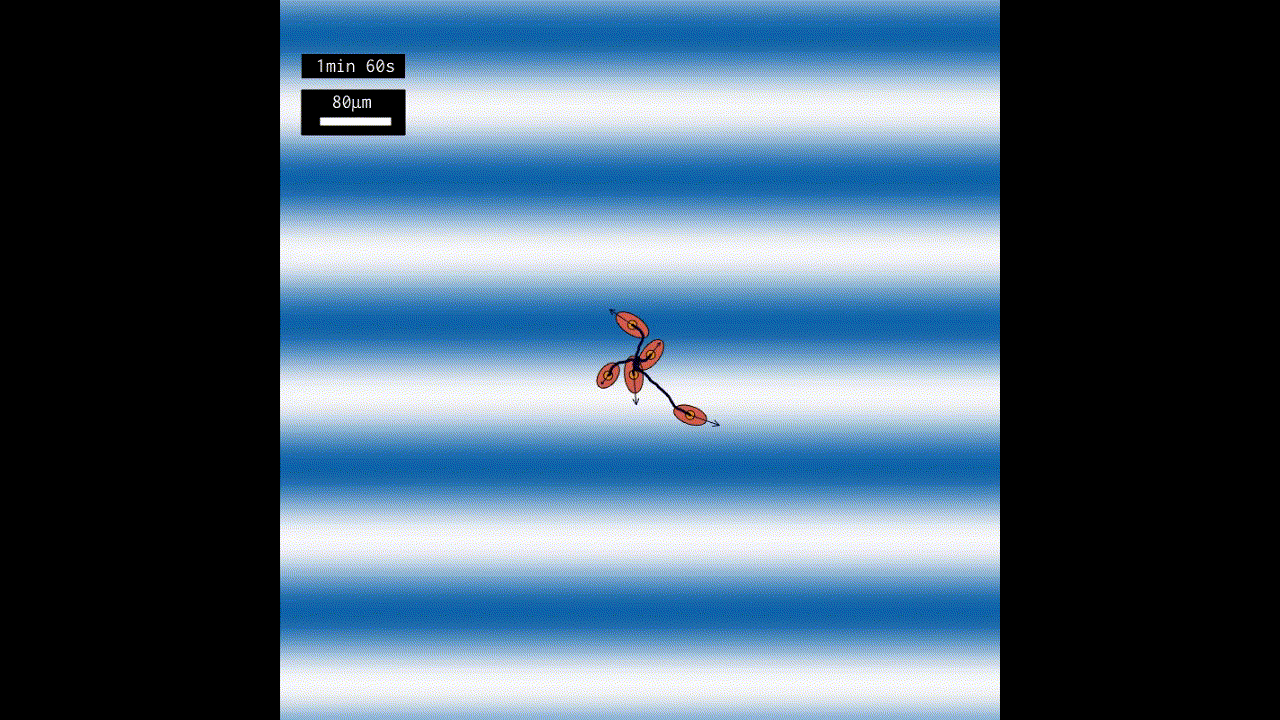

Supplement: Supplementary file 9 — Supplementary Information 9. [file 41598_2025_2804_MOESM9_ESM.gif]

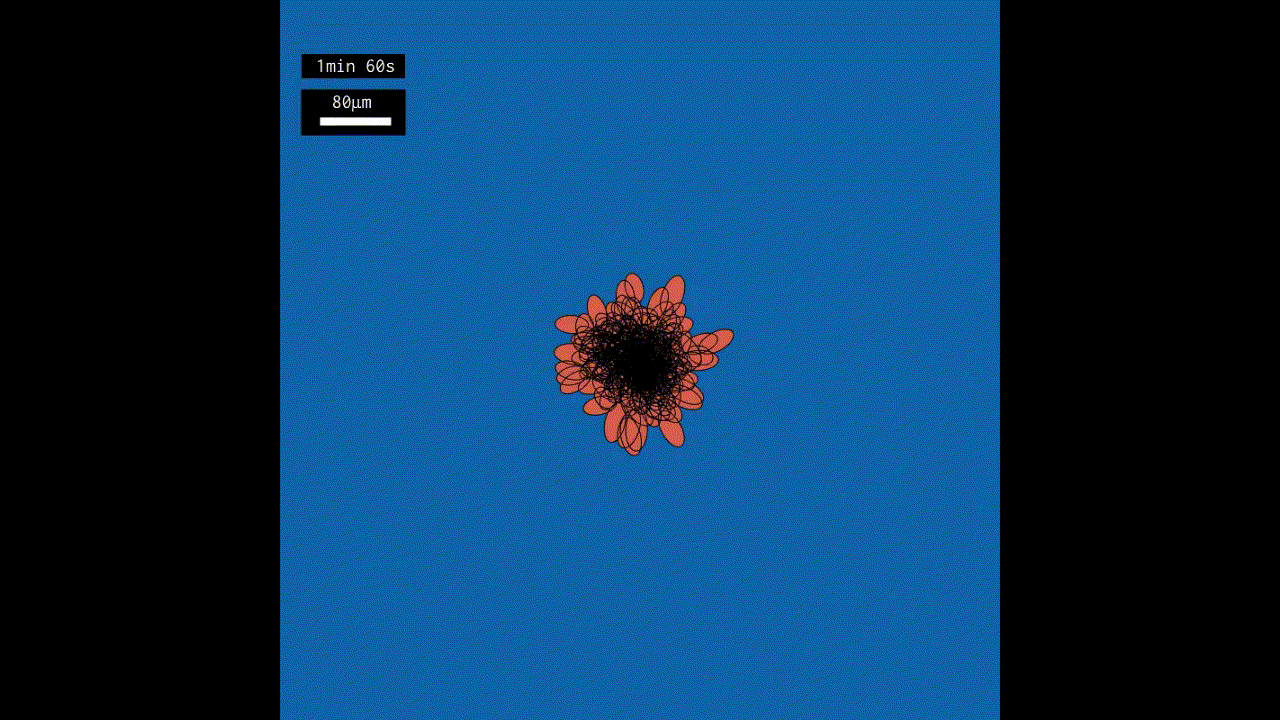

Supplement: Supplementary file 10 — Supplementary Information 10. [file 41598_2025_2804_MOESM10_ESM.gif]

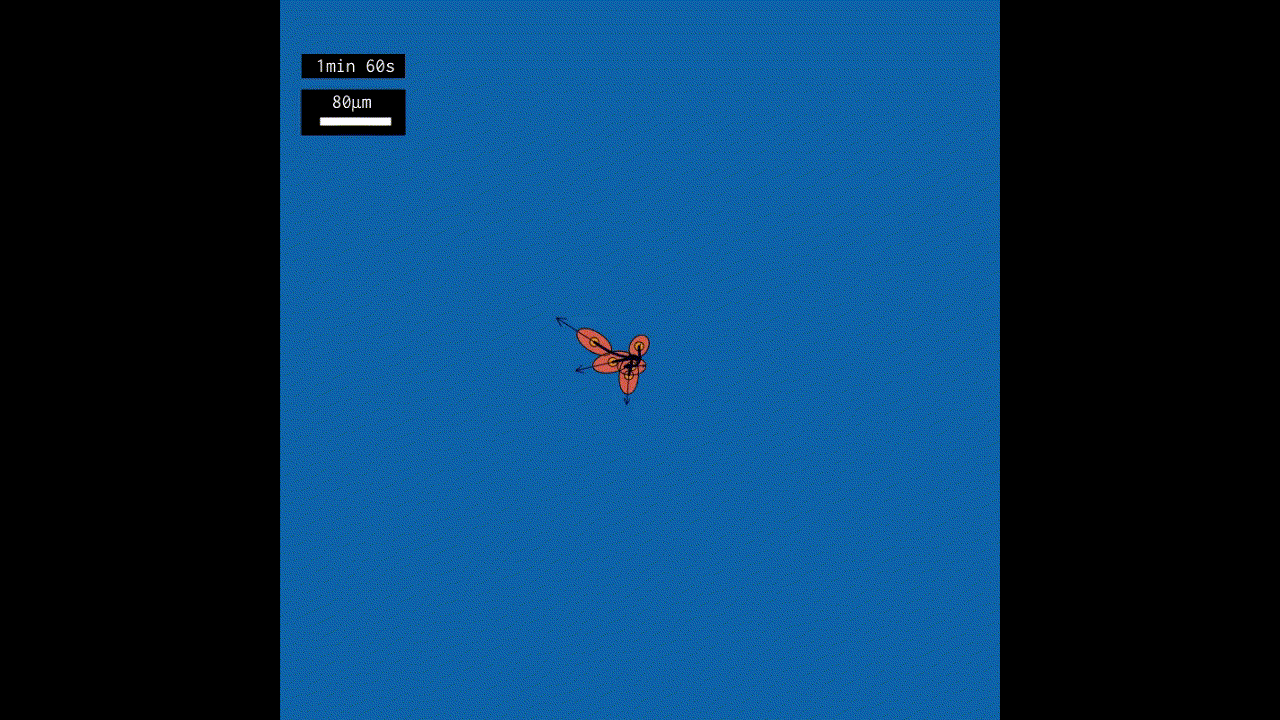

Supplement: Supplementary file 11 — Supplementary Information 11. [file 41598_2025_2804_MOESM11_ESM.gif]
